# Supplementary material for: The dynamics and strategy of RNA replication in astroviruses
Source: NAR Mol Med. 2026 Apr 20;3(2):ugag021. doi: 10.1093/narmme/ugag021 (PMC13136897; doi:10.1093/narmme/ugag021)
Supplement: ugag021_Supplemental_File [file ugag021_supplemental_file.docx]

Supplementary information file

The dynamics and strategy of RNA replication in astroviruses

David Noyvert^1^, Imran M. Darr^1^, Ksenia Fominykh^1^, Jacqueline Hankinson^1^, Nina Lukhovitskaya^1^, Andrew E. Firth^1^*, Valeria Lulla^1^*

^1^ Department of Pathology, University of Cambridge, Cambridge, United Kingdom

*** Correspondence:** Andrew E. Firth, [aef24@cam.ac.uk](mailto:aef24@cam.ac.uk); Valeria Lulla, [vl284@cam.ac.uk](mailto:vl284@cam.ac.uk)

**Supplementary Figure S1. Establishment of strand-specific RT-qPCR.** Each qPCR primer pair was tested using 10-fold dilutions of T7 transcripts corresponding to the indicated strand (filled circles). As a strand specificity control, each dilution was mixed with 10^8^ genome copies of the opposite strand (“+10^8^ opp”, white rectangles). Primer efficiency (PE) was calculated using the formula PE = 100×(10^(1/slope)^-1) and ranged between 95-105%. No significant changes were detected for opposite-strand-containing samples.

**
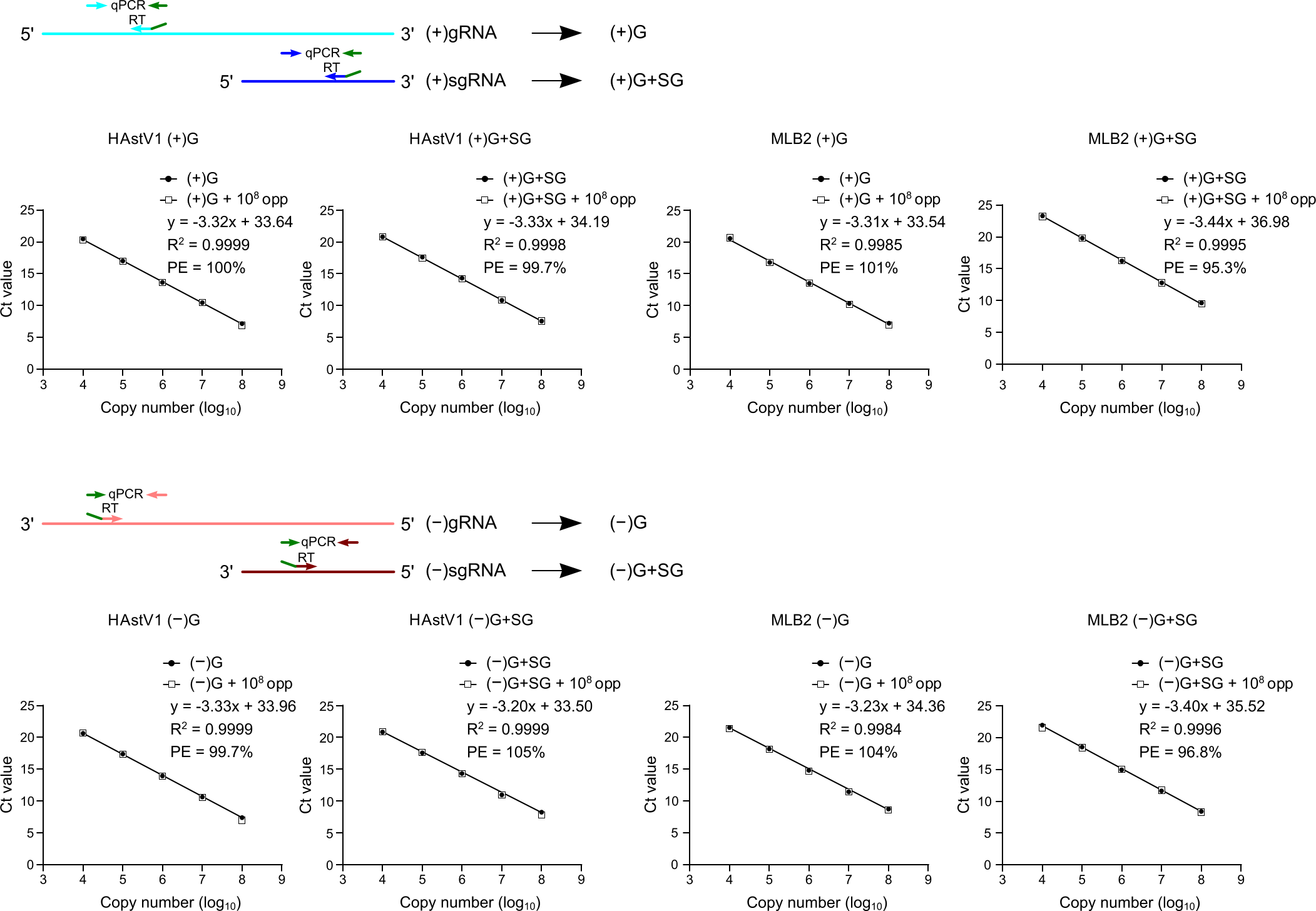
**

**Supplementary Figure S2. Read-length distributions of RNA-seq libraries.** Length distribution of virus-mapping fragments, after σ-clipping, for the first (**A**), second (**B**) and third (**C**) high throughput sequencing datasets.

**A**


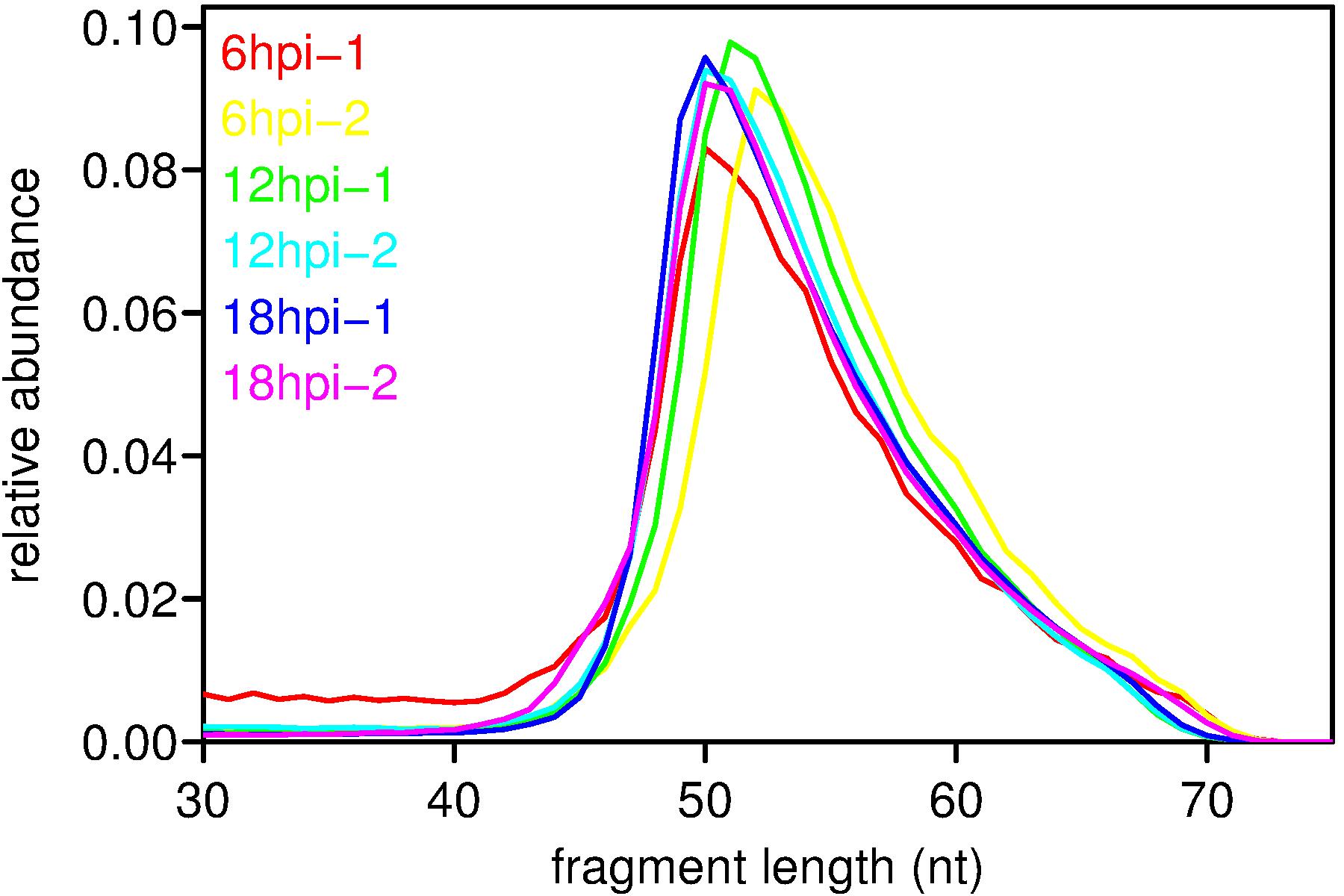


**B**

**
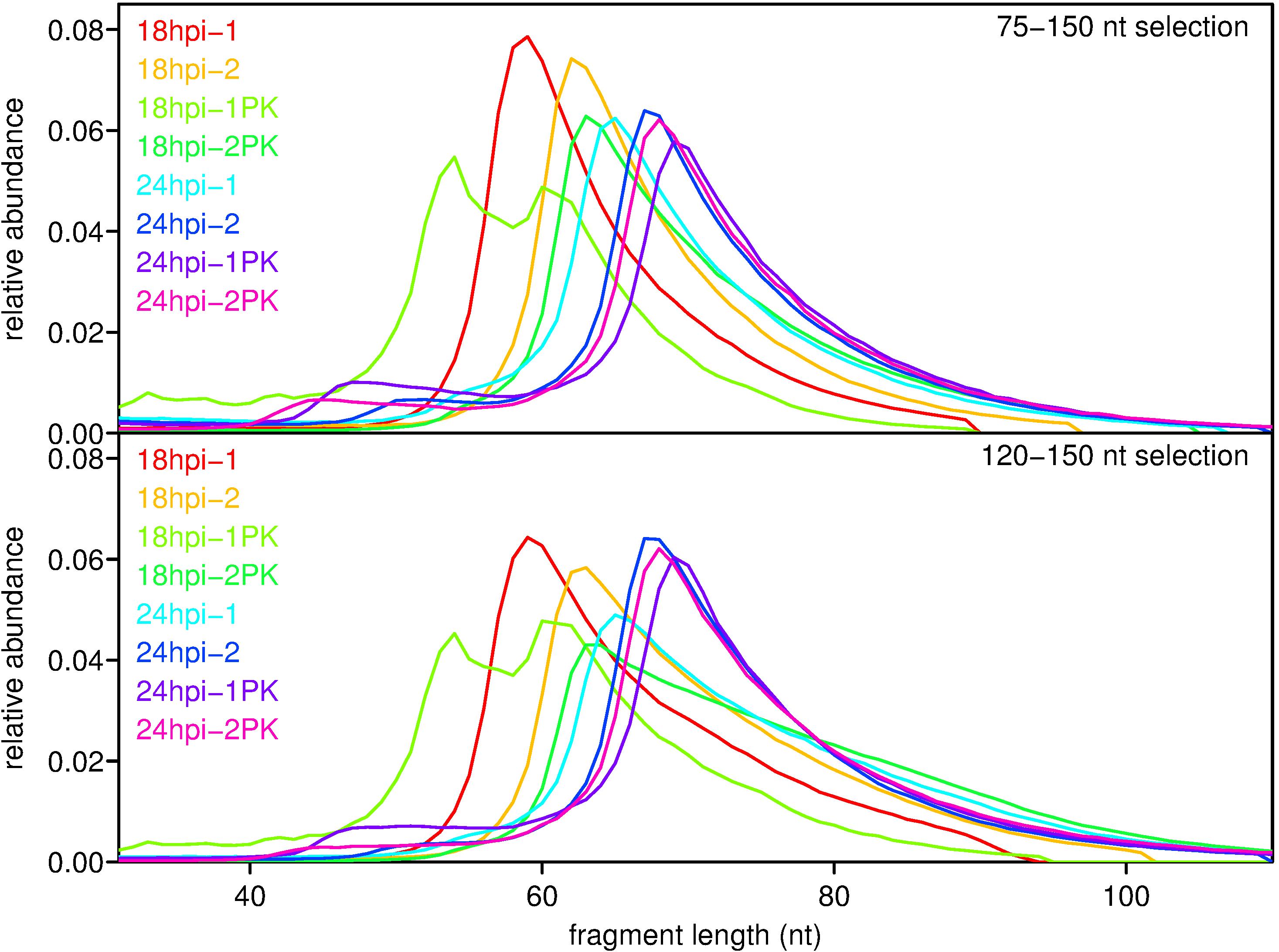
**

**C**

**
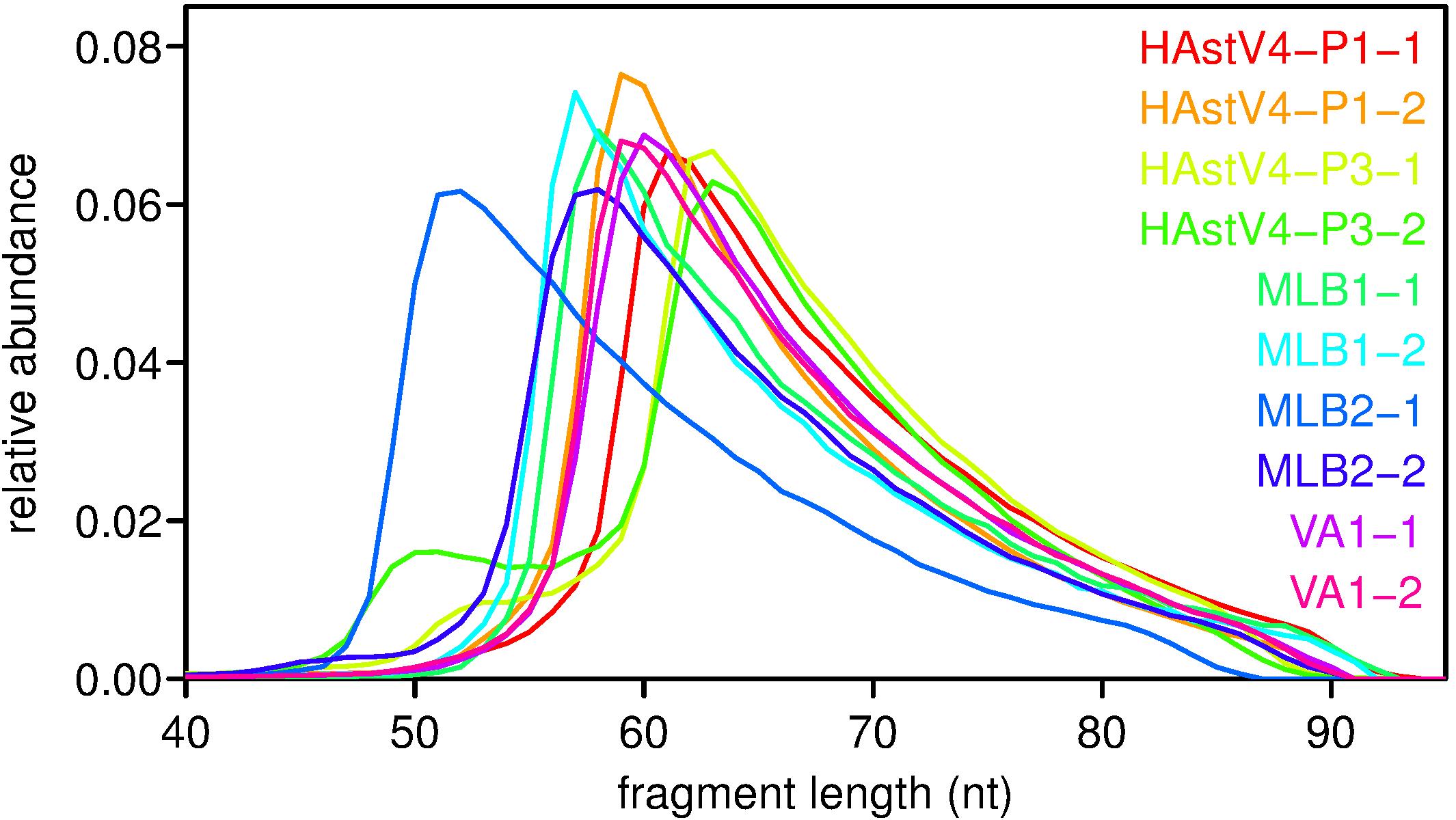
**

**Supplementary Figure S3. Total coverage of vRNA(+) and vRNA(−).** Caco-2 cells were infected with HAstV1 at MOI 5 and harvested at 6, 12 or 18 hpi in duplicate. Fragments were mapped to vRNA(+) or vRNA(−), and total depth of coverage summed. The y-axis scale is arbitrary but vRNA(−) coverage depth is scaled relative to vRNA(+) coverage depth by the indicated factor to aid visualization.

**
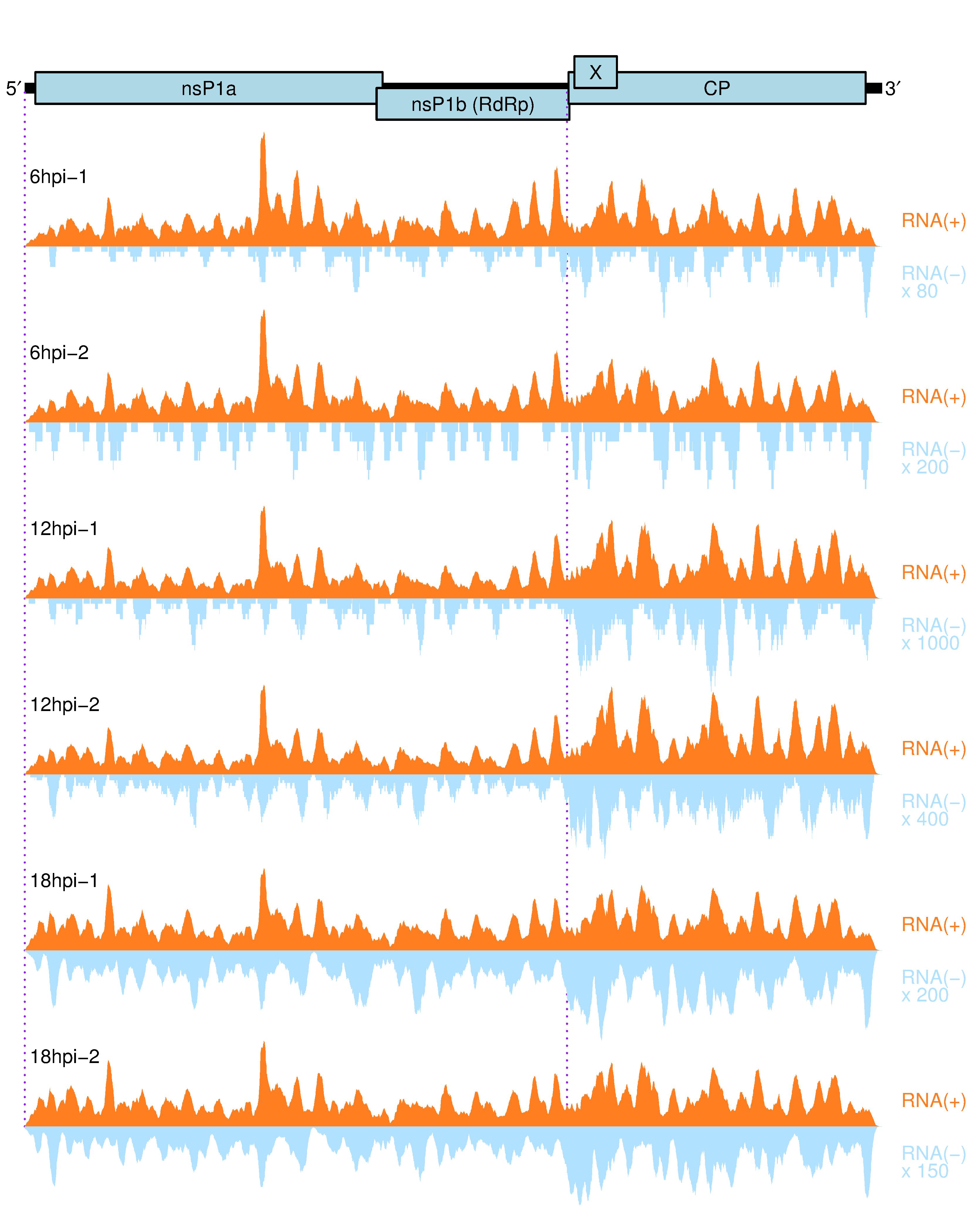
**

**Supplementary Figure S4. Histograms showing positions of 5′ ends of fragments mapping to vRNA(+).** Caco-2 cells were infected with HAstV1 at MOI 5 and harvested at 6, 12 or 18 hpi in duplicate. Counts are normalized to fragments per million fragments mapped to vRNA(+) or host mRNA(+) (FPM).

**
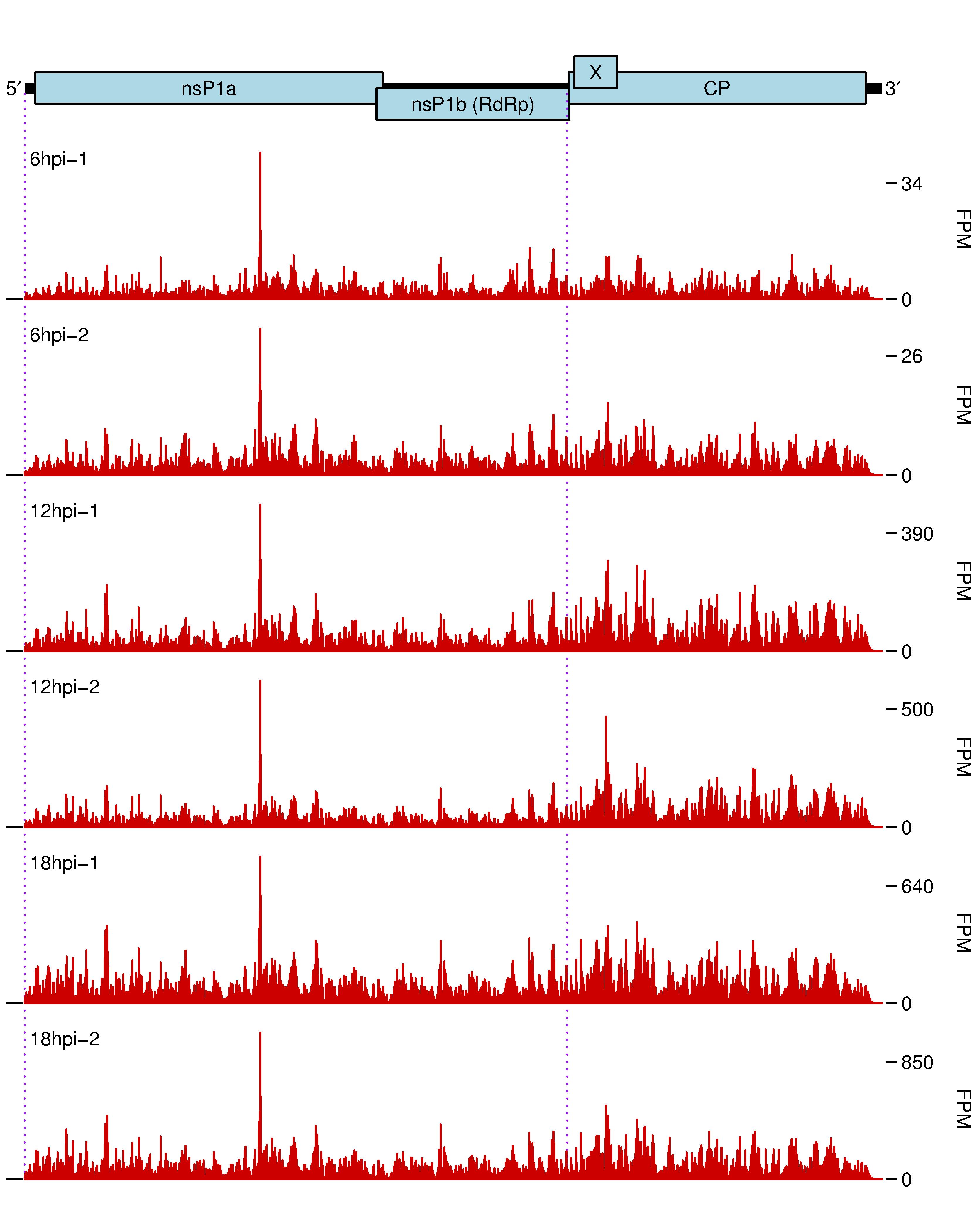
**

**Supplementary Figure S5. Histograms showing positions of 3′ ends of fragments mapping to vRNA(−).** Caco-2 cells were infected with HAstV1 at MOI 5 and harvested at 6, 12 or 18 hpi in duplicate. Counts are normalized to fragments per million fragments mapped to vRNA(+) or host mRNA(+) (FPM). Histograms show 3′ ends of negative-sense fragments, corresponding to 5′ ends of the positive-sense reverse complements of the fragments.

**
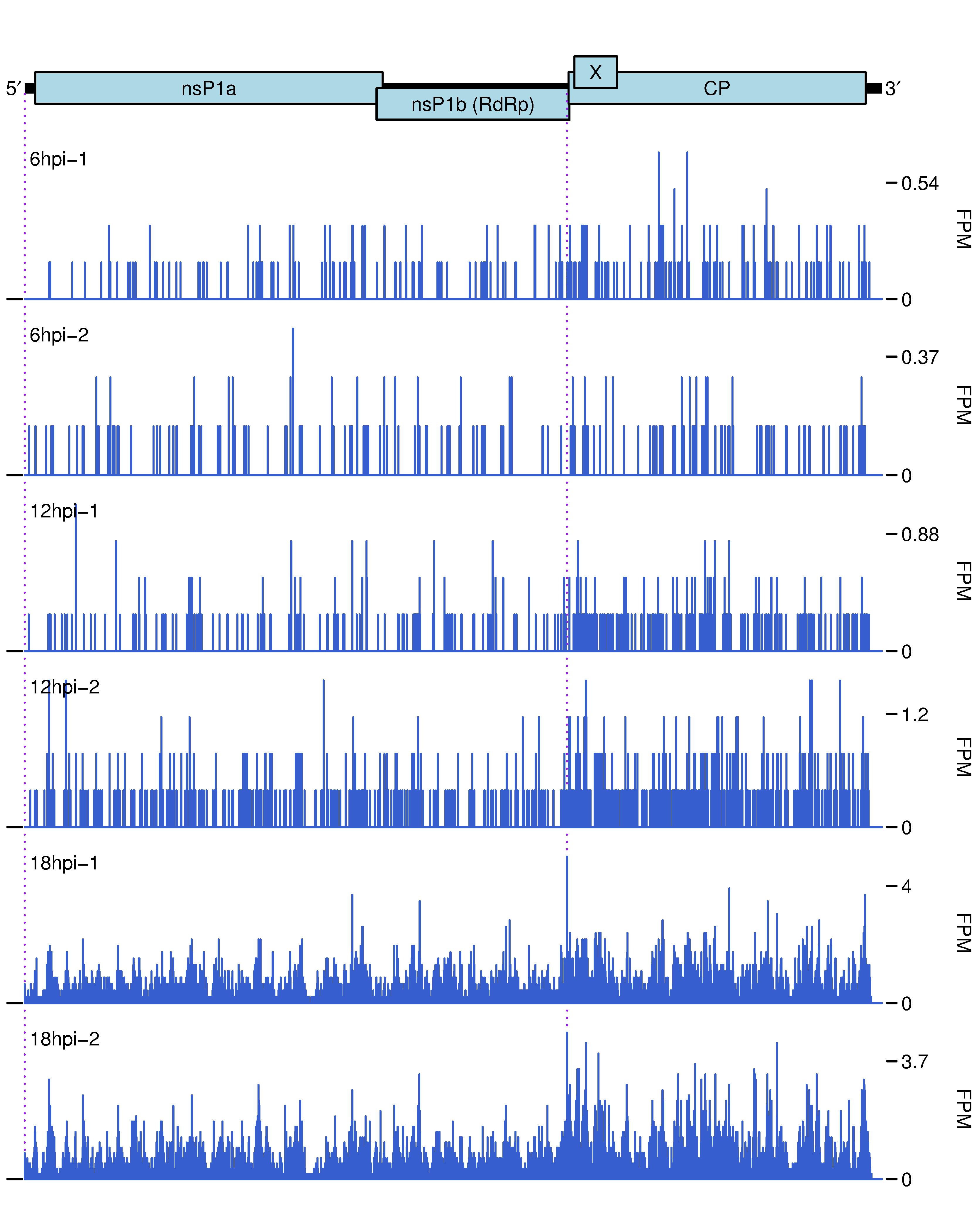
**

**Supplementary Figure S6. Histograms showing positions of 5′ ends of fragments mapping to vRNA(+).** Caco-2 cells were infected with HAstV1 at MOI 5 and harvested at 18 or 24 hpi in duplicate, with or without proteinase K (PK) treatment; nominally 75–150 nt fragments were selected for sequencing. Counts are normalized to fragments per million fragments mapped to vRNA(+) or host mRNA(+) (FPM).

**
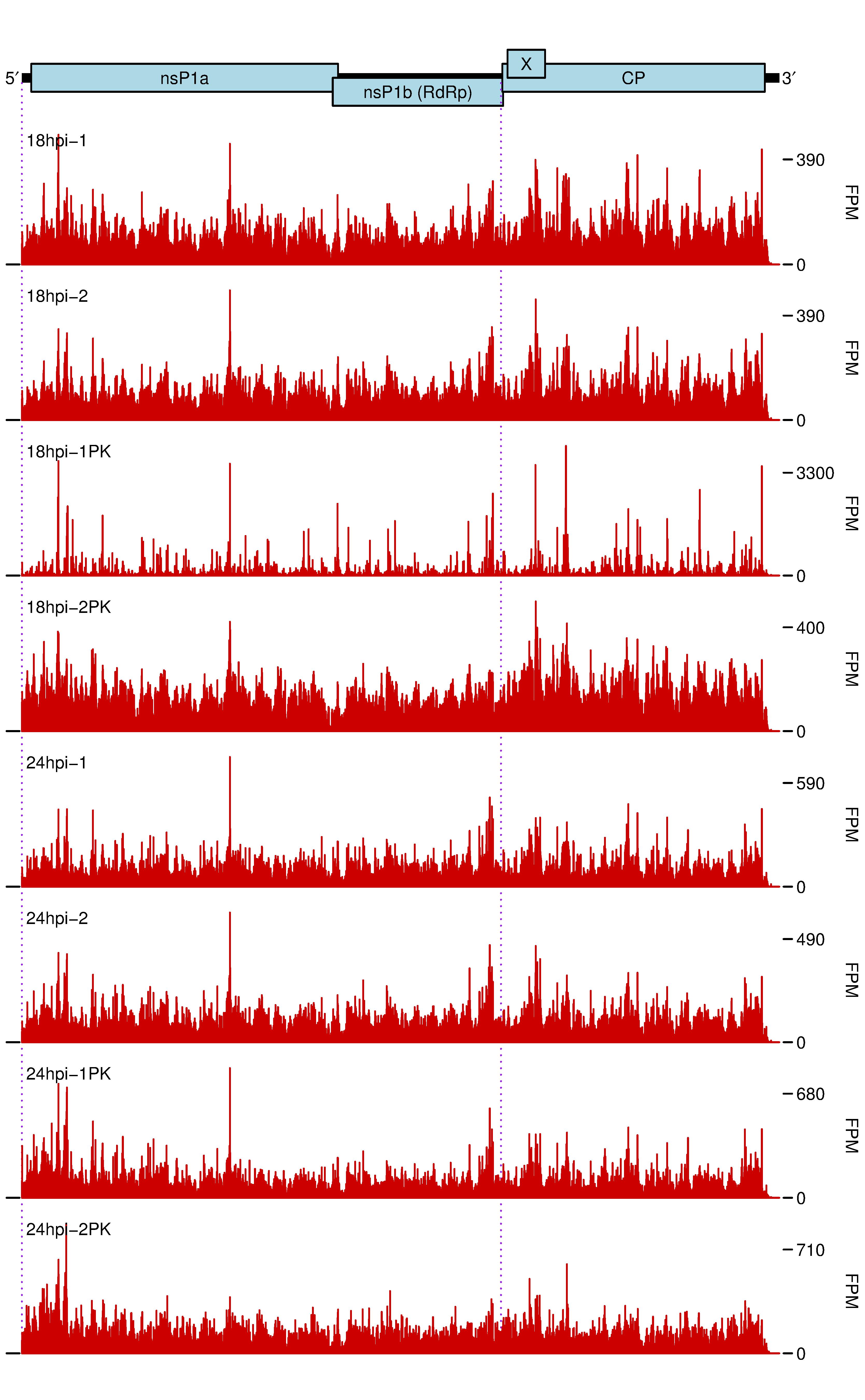
**

**
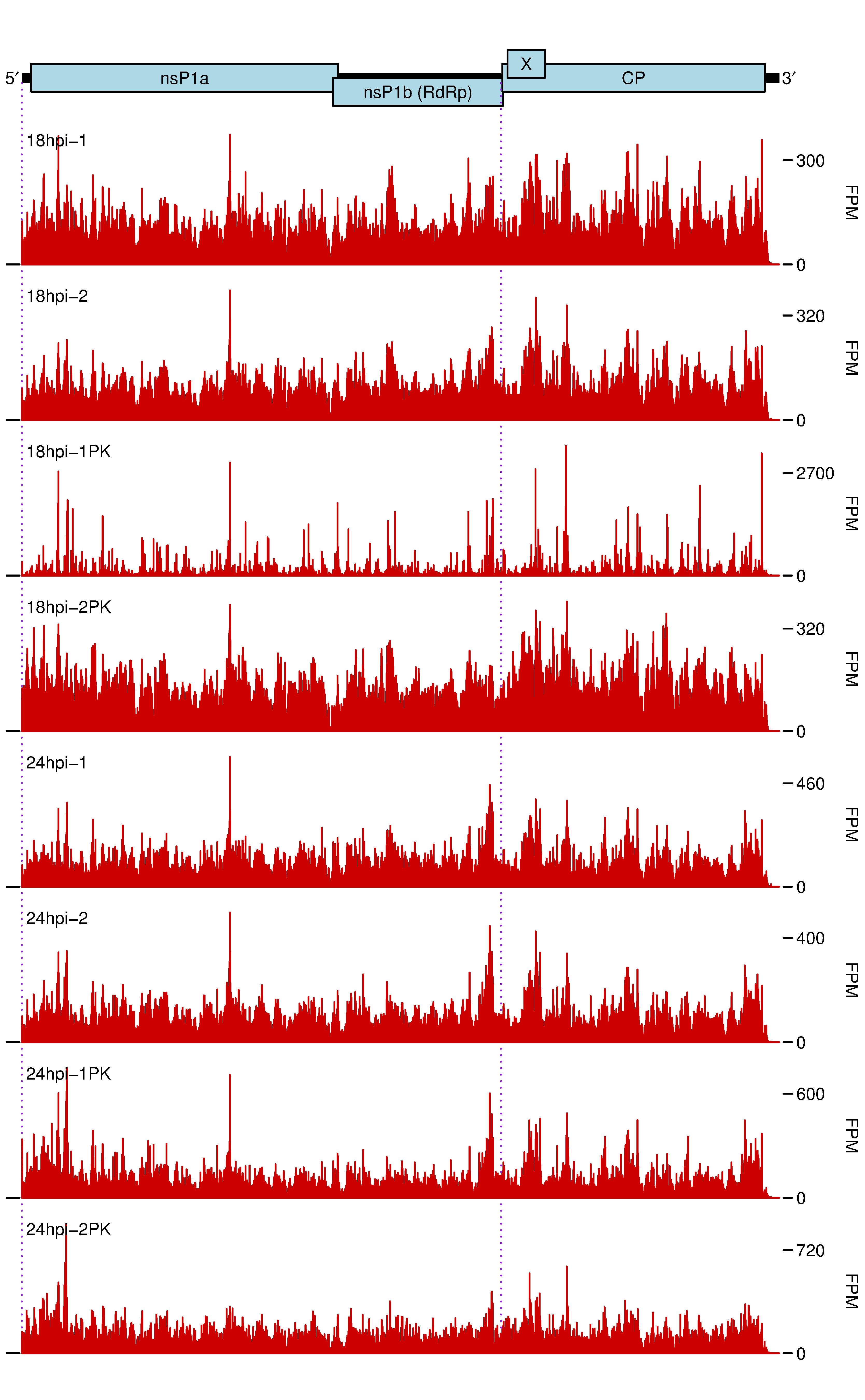
Supplementary Figure S7. Histograms showing positions of 5′ ends of fragments mapping to vRNA(+).** Caco-2 cells were infected with HAstV1 at MOI 5 and harvested at 18 or 24 hpi in duplicate, with or without proteinase K (PK) treatment; nominally 120–150 nt fragments were selected for sequencing. Counts are normalized to fragments per million fragments mapped to vRNA(+) or host mRNA(+) (FPM).

**Supplementary Figure S8. Levels of astrovirus positive/negative-sense gRNA/sgRNA species.** Caco-2 cells were infected with HAstV1 at MOI 5 and harvested at 18 or 24 hpi in duplicate, with or without proteinase K (PK) treatment. (**A**) Bar graphs showing the density of mapped fragments in the sgRNA region (pink), outside of the sgRNA region (red) and the difference (yellow). Coverage depth was quantified as fragments per kilobase per million fragments mapped to vRNA(+) or host mRNA(+). Fragments mapping to the sgRNA region may derive from either gRNA or sgRNA; the difference (yellow) in density between the sgRNA and non-sgRNA regions was used to estimate the relative abundance of sgRNA, whereas the density in the non-sgRNA region (red) was used to estimate the relative abundance of gRNA. (**B**) Relative densities of (+)gRNA, (+)sgRNA, (−)gRNA and (−)sgRNA. Numbers below bars show the estimated sgRNA:gRNA ratio (1 d.p.). (**C**) Estimated (−):(+) ratio for gRNA and sgRNA species. The (−):(+) sgRNA ratios are not shown for the 24 hpi samples because the (+)sgRNAvalues are unreliable at this time point (insufficient RNA-seq density difference between the gRNA/sgRNA overlap region and the gRNA-only region).

**A**

**
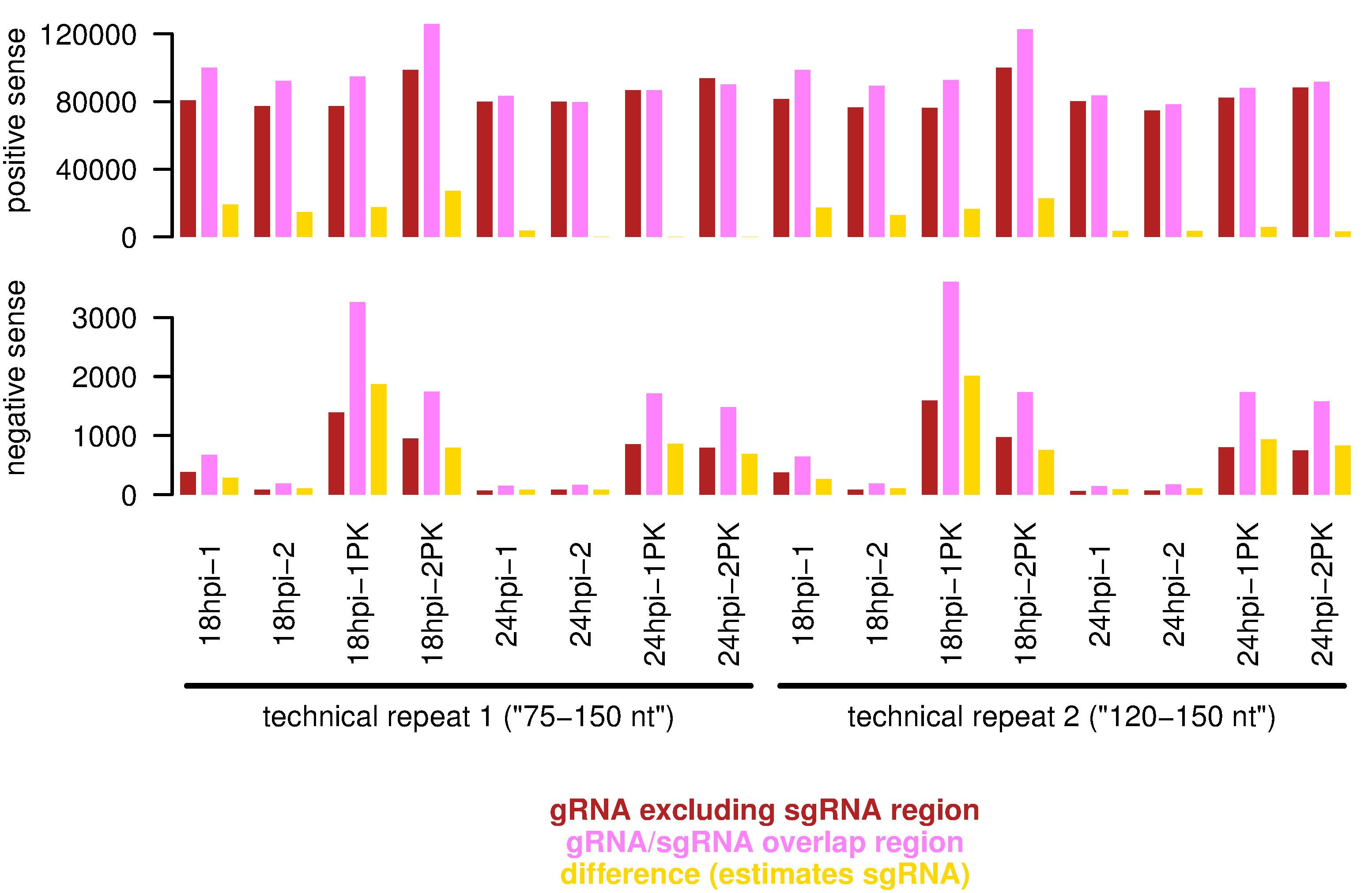
**

**B C**

**
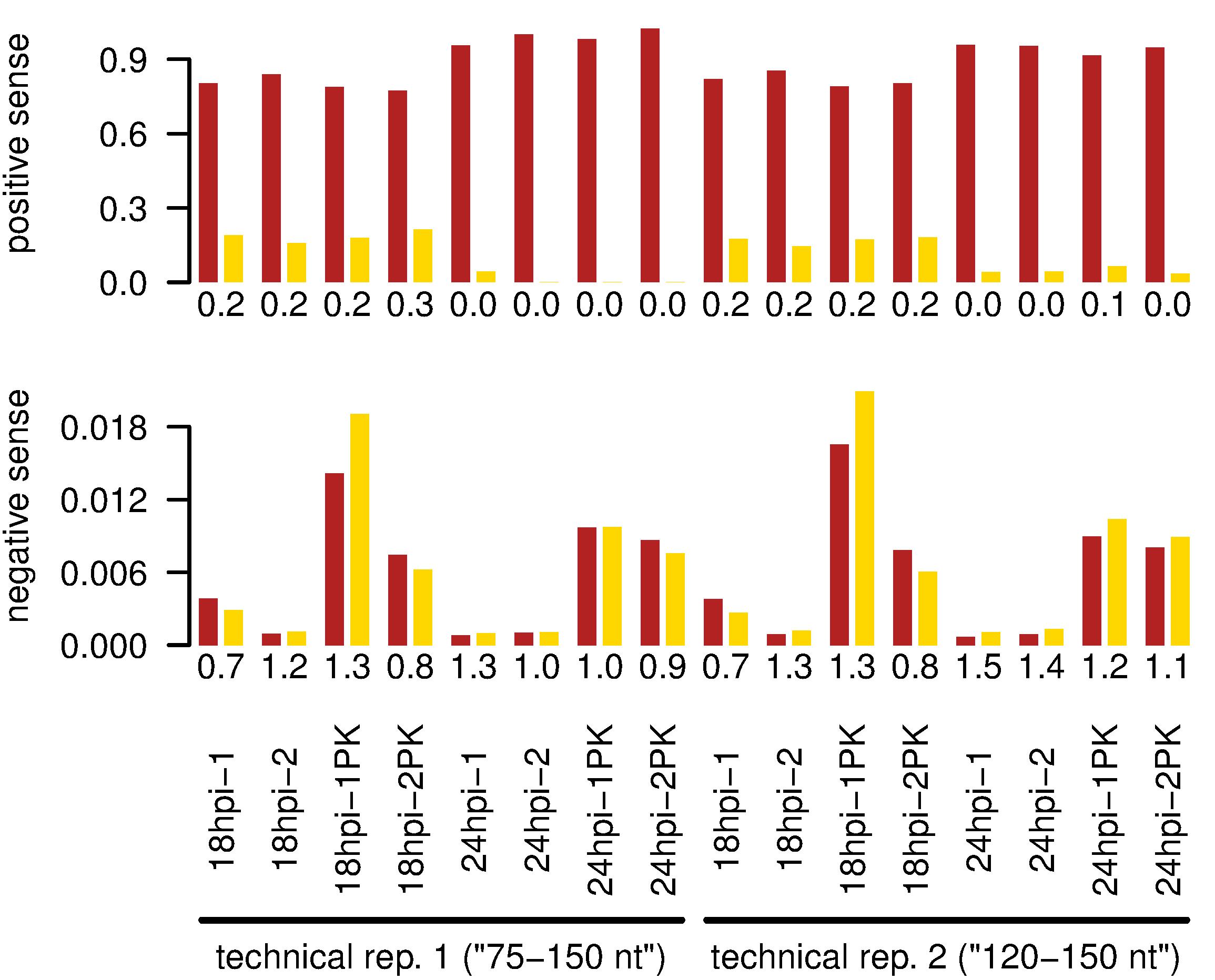

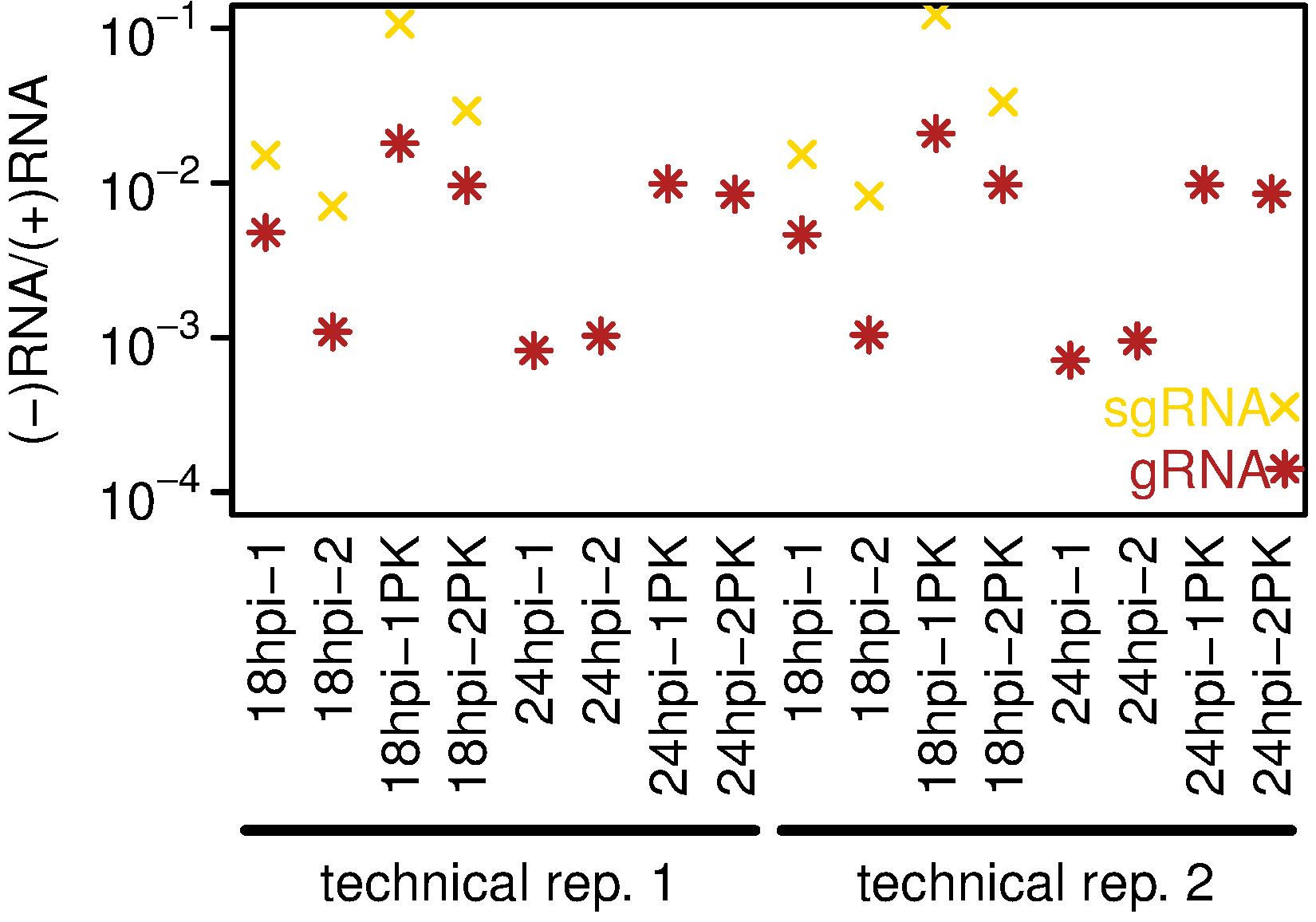
**

**Supplementary Figure S9. Total coverage of vRNA(+) and vRNA(−).** Caco-2 cells were infected with HAstV1 at MOI 5 and harvested at 18 or 24 hpi in duplicate, with or without proteinase K (PK) treatment; nominally 75–150 nt fragments were selected for sequencing. Fragments were mapped to vRNA(+) or vRNA(−), and total depth of coverage summed. The y-axis scale is arbitrary but vRNA(−) coverage depth is scaled relative to vRNA(+) coverage depth by the indicated factor to aid visualization.

**
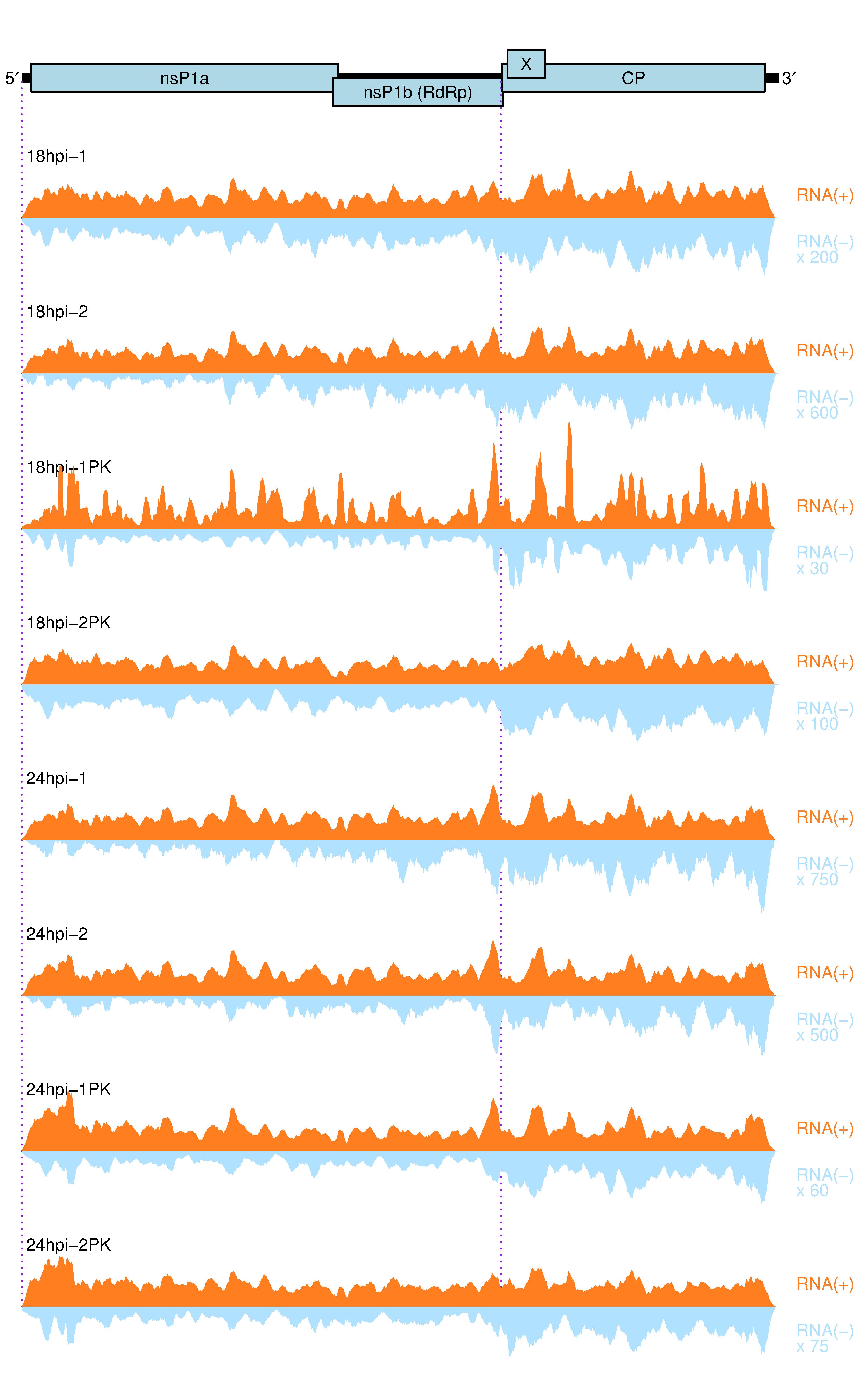
**

**
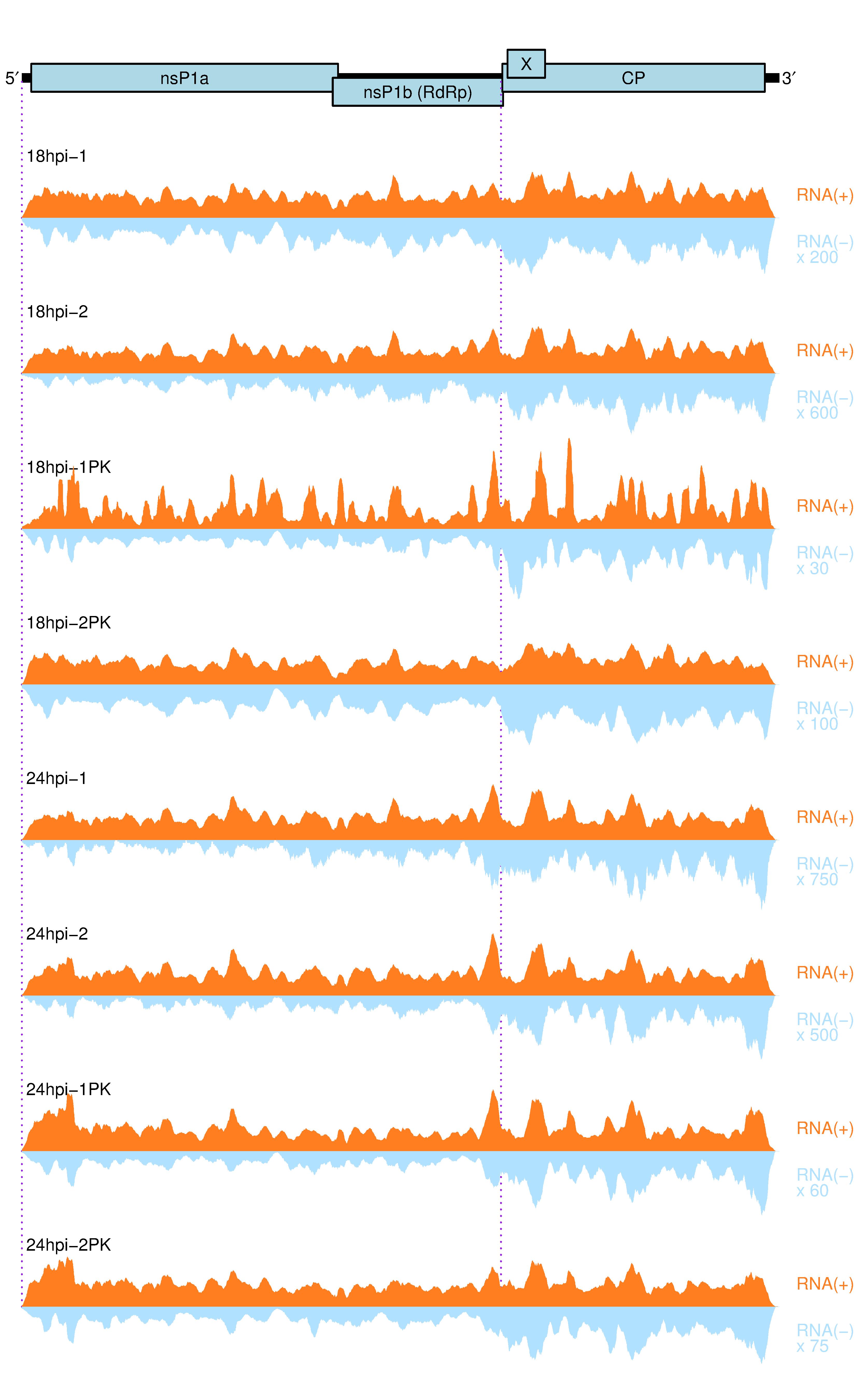
Supplementary Figure S10. Total coverage of vRNA(+) and vRNA(−).** Caco-2 cells were infected with HAstV1 at MOI 5 and harvested at 18 or 24 hpi in duplicate, with or without proteinase K (PK) treatment; nominally 120–150 nt fragments were selected for sequencing. Fragments were mapped to vRNA(+) or vRNA(−), and total depth of coverage summed. The y-axis scale is arbitrary but vRNA(−) coverage depth is scaled relative to vRNA(+) coverage depth by the indicated factor to aid visualization.

**Supplementary Figure S11. Histograms showing positions of 3′ ends of fragments mapping to vRNA(−).** Caco-2 cells were infected with HAstV1 at MOI 5 and harvested at 18 or 24 hpi in duplicate, with or without proteinase K (PK) treatment; nominally 75–150 nt fragments were selected for sequencing. Counts are normalized to fragments per million fragments mapped to vRNA(+) or host mRNA(+) (FPM). Histograms show 3′ ends of negative-sense fragments, corresponding to 5′ ends of the positive-sense reverse complements of the fragments.

**
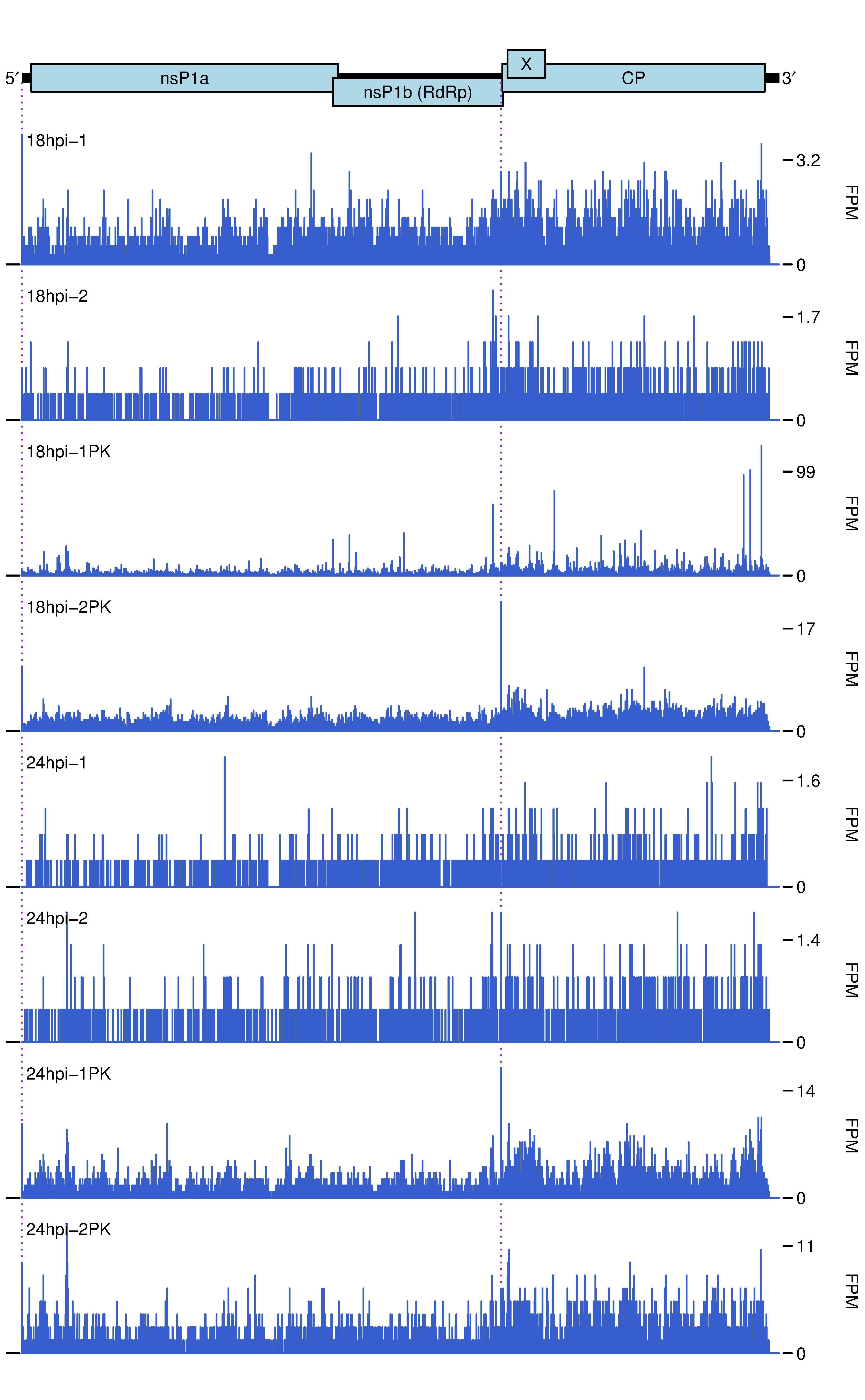
**

**Supplementary Figure S12. Histograms showing positions of 3′ ends of fragments mapping to vRNA(−).** Caco-2 cells were infected with HAstV1 at MOI 5 and harvested at 18 or 24 hpi in duplicate, with or without proteinase K (PK) treatment; nominally 120–150 nt fragments were selected for sequencing. Counts are normalized to fragments per million fragments mapped to vRNA(+) or host mRNA(+) (FPM). Histograms show 3′ ends of negative-sense fragments, corresponding to 5′ ends of the positive-sense reverse complements of the fragments.

**
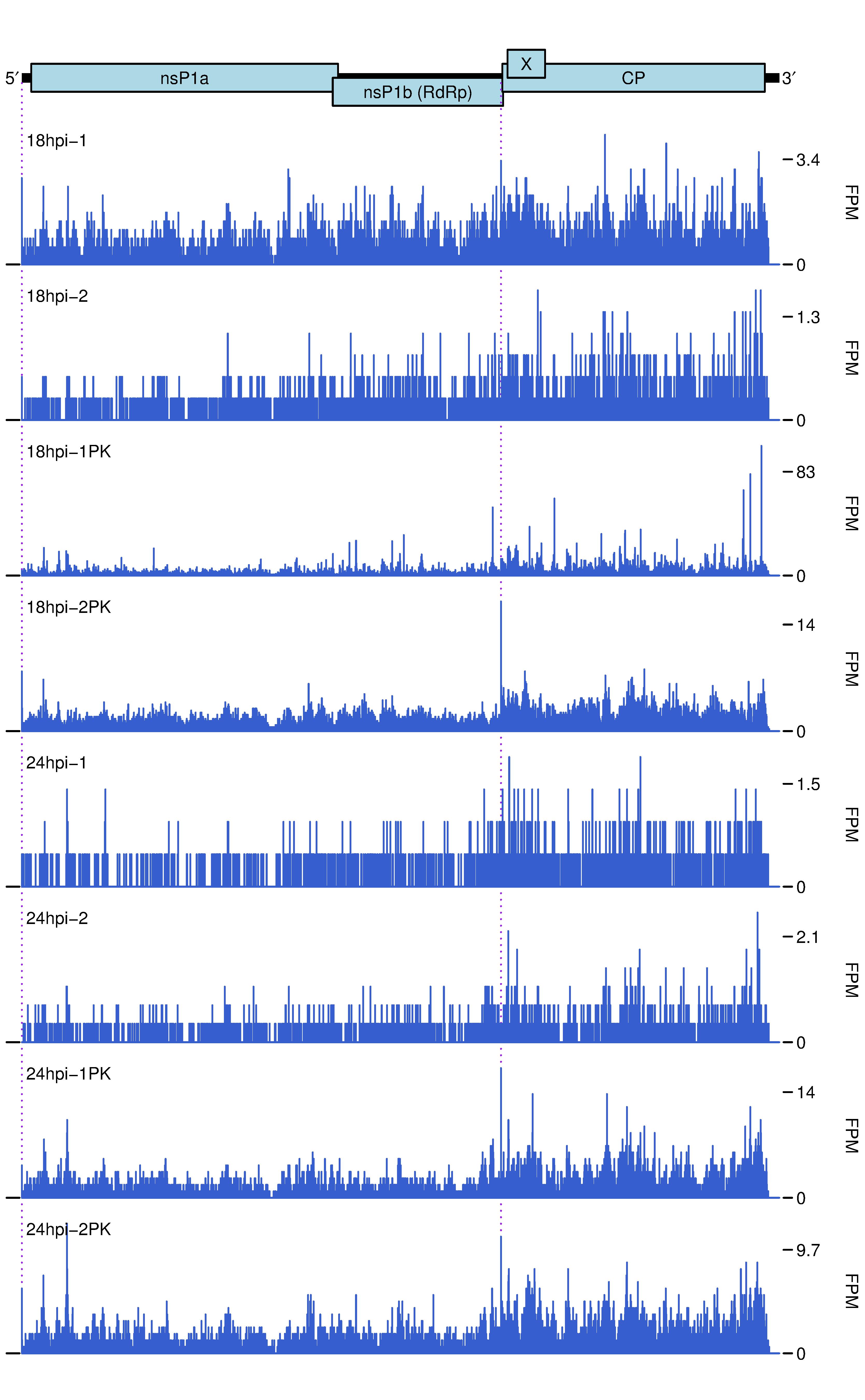
**

**Supplementary Figure S13. Levels of astrovirus positive/negative-sense gRNA/sgRNA species.** Cells were infected with HAstV4 (in quadruplicate), or MLB1, MLB2 or VA1 (in duplicate) viruses at MOI 5 and harvested at 24 hpi Caco-2 cells were used for HAstV4 and VA1, and Huh7.5.1 cells were used for MLB1 and MLB2. **(A)** Bar graphs showing the density of mapped fragments in the sgRNA region (pink), outside of the sgRNA region (red) and the difference (yellow). Coverage depth was quantified as fragments per kilobase per million fragments mapped to vRNA(+) or host mRNA(+). Fragments mapping to the sgRNA region may derive from either gRNA or sgRNA; the difference (yellow) in density between the sgRNA and non-sgRNA regions was used to estimate the relative abundance of sgRNA, whereas the density in the non-sgRNA region (red) was used to estimate the relative abundance of gRNA. (**B**) Relative densities of (+)gRNA, (+)sgRNA, (−)gRNA and (−)sgRNA. Numbers below bars show the estimated sgRNA:gRNA ratio (1 d.p.). (**C**) Estimated (−):(+) ratio for gRNA and sgRNA species.

**A**

**
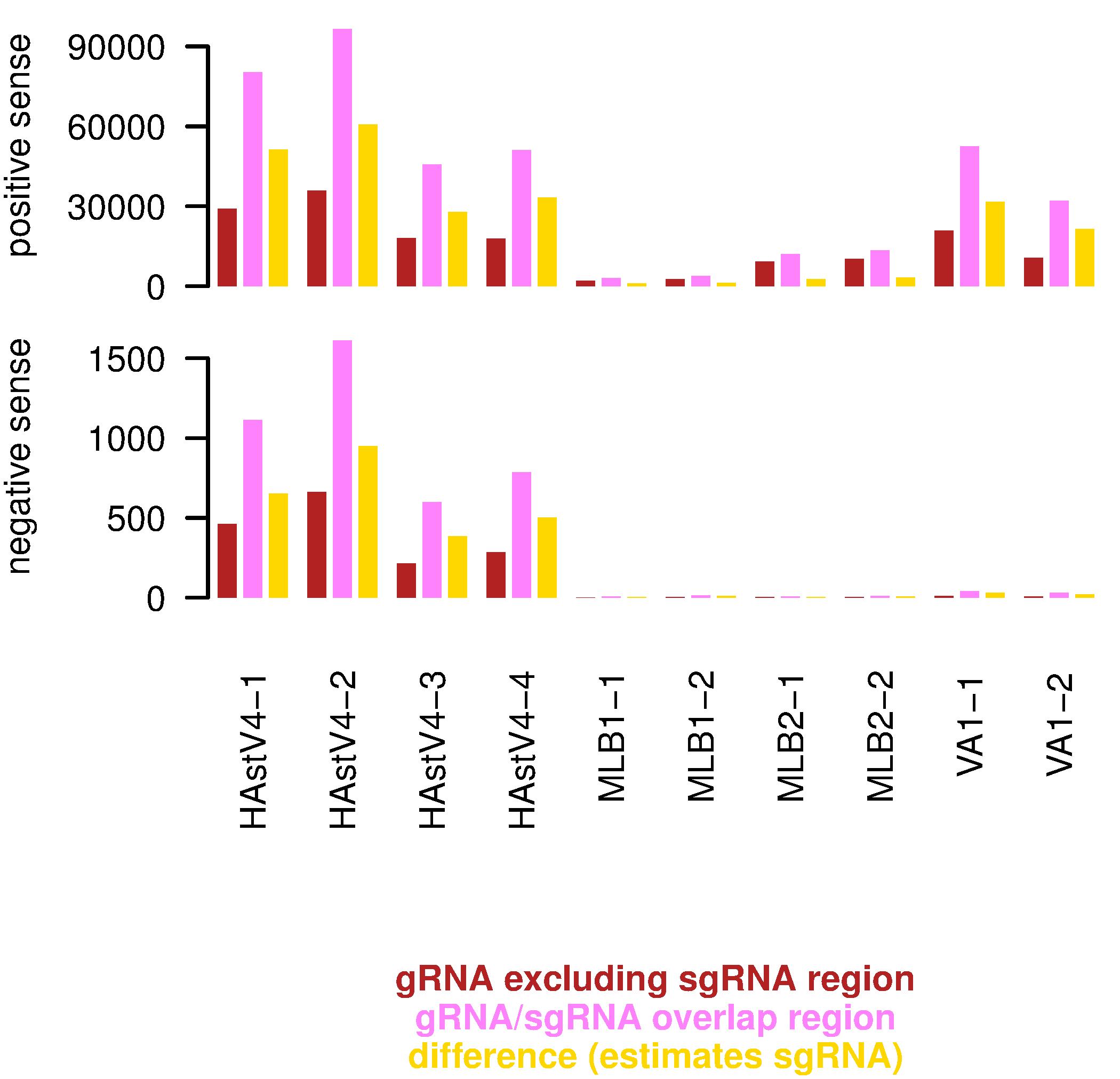
**

**B C**

**
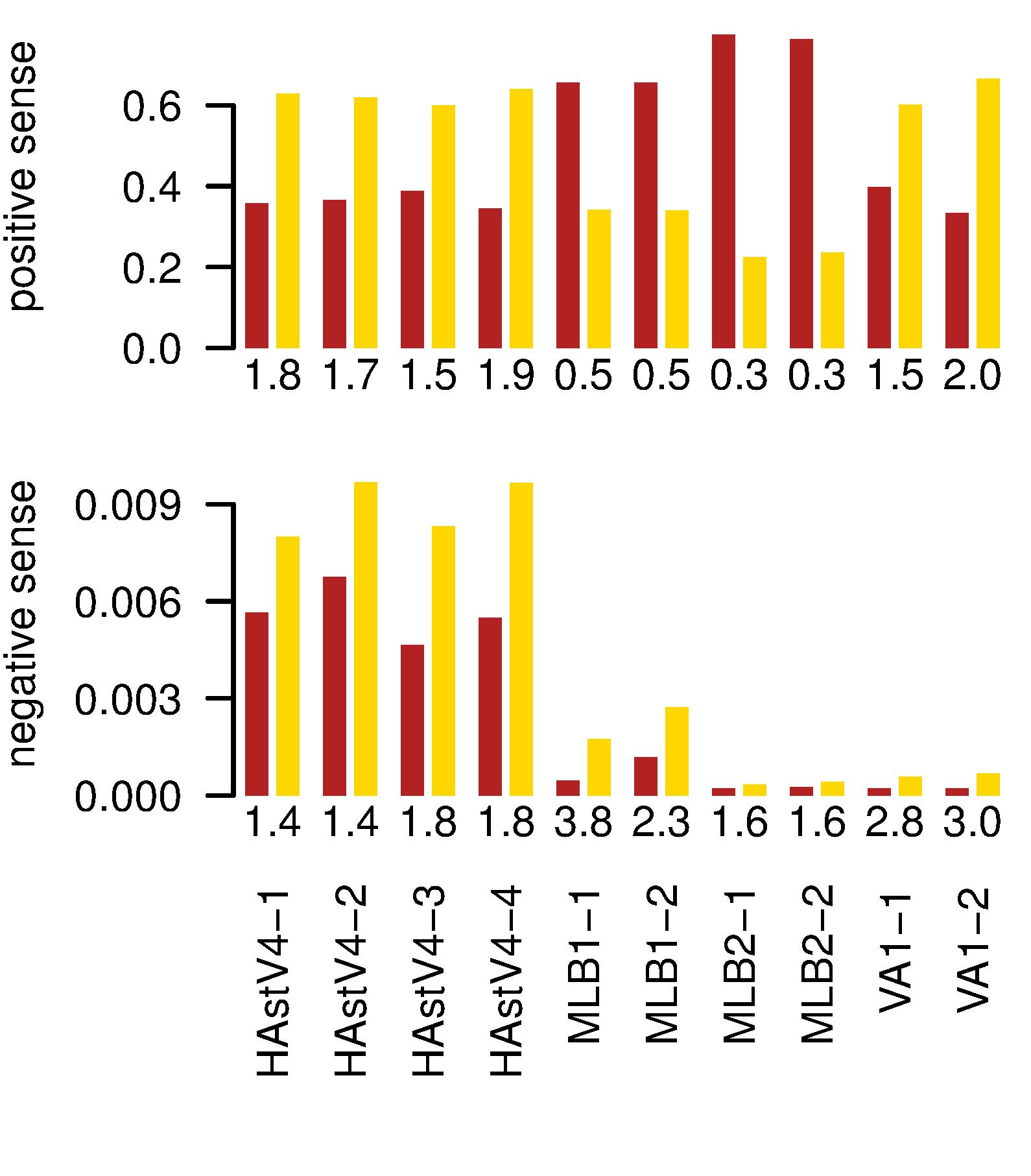

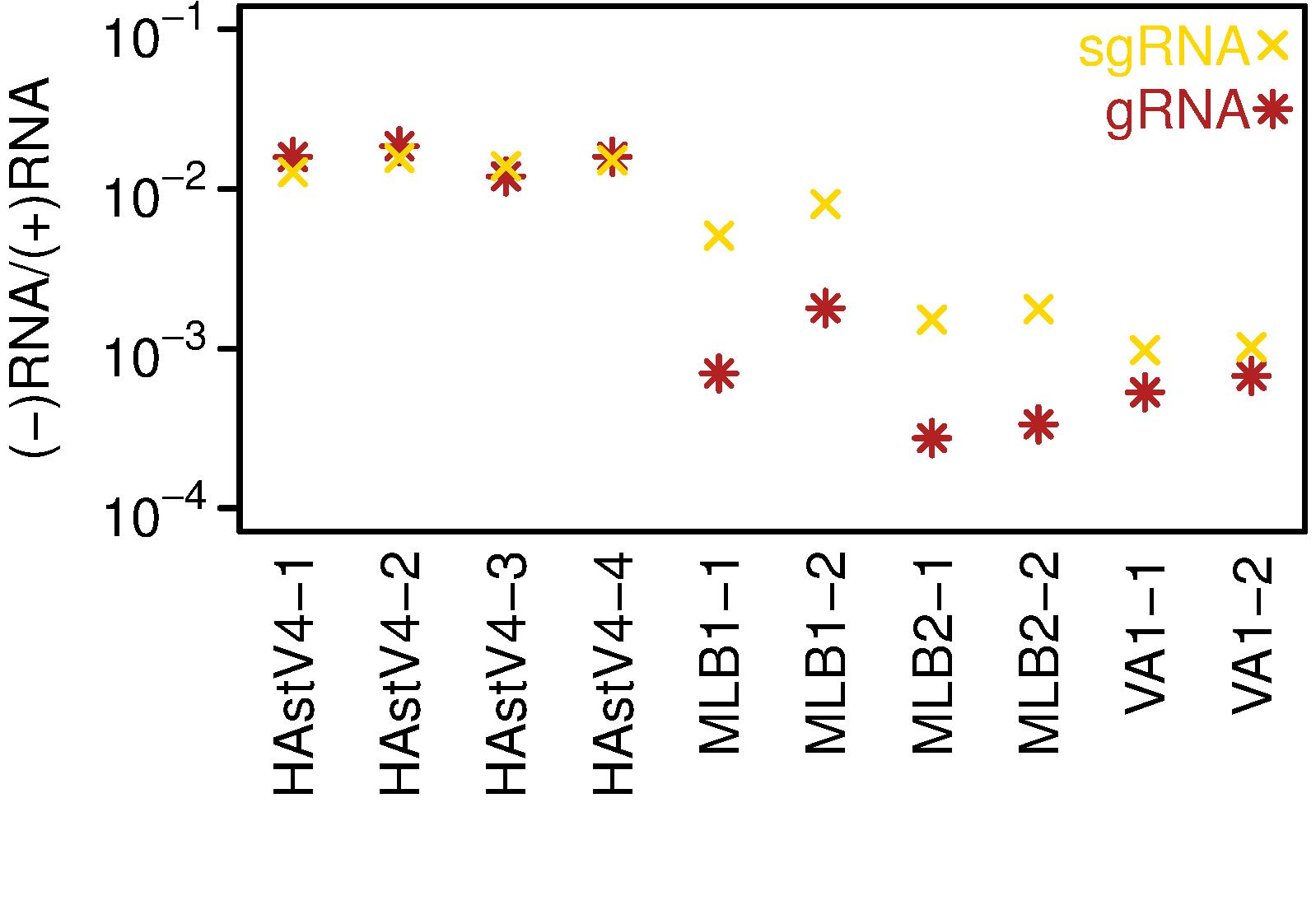
**

**Supplementary Figure S14. Total coverage of vRNA(+) and vRNA(−).** Caco-2 cells were infected with HAstV4 at MOI 5 and harvested at 24 hpi in quadruplicate. Fragments were mapped to vRNA(+) or vRNA(−), and total depth of coverage summed. The y-axis scale is arbitrary but vRNA(−) coverage depth is scaled relative to vRNA(+) coverage depth by the indicated factor to aid visualization.

**
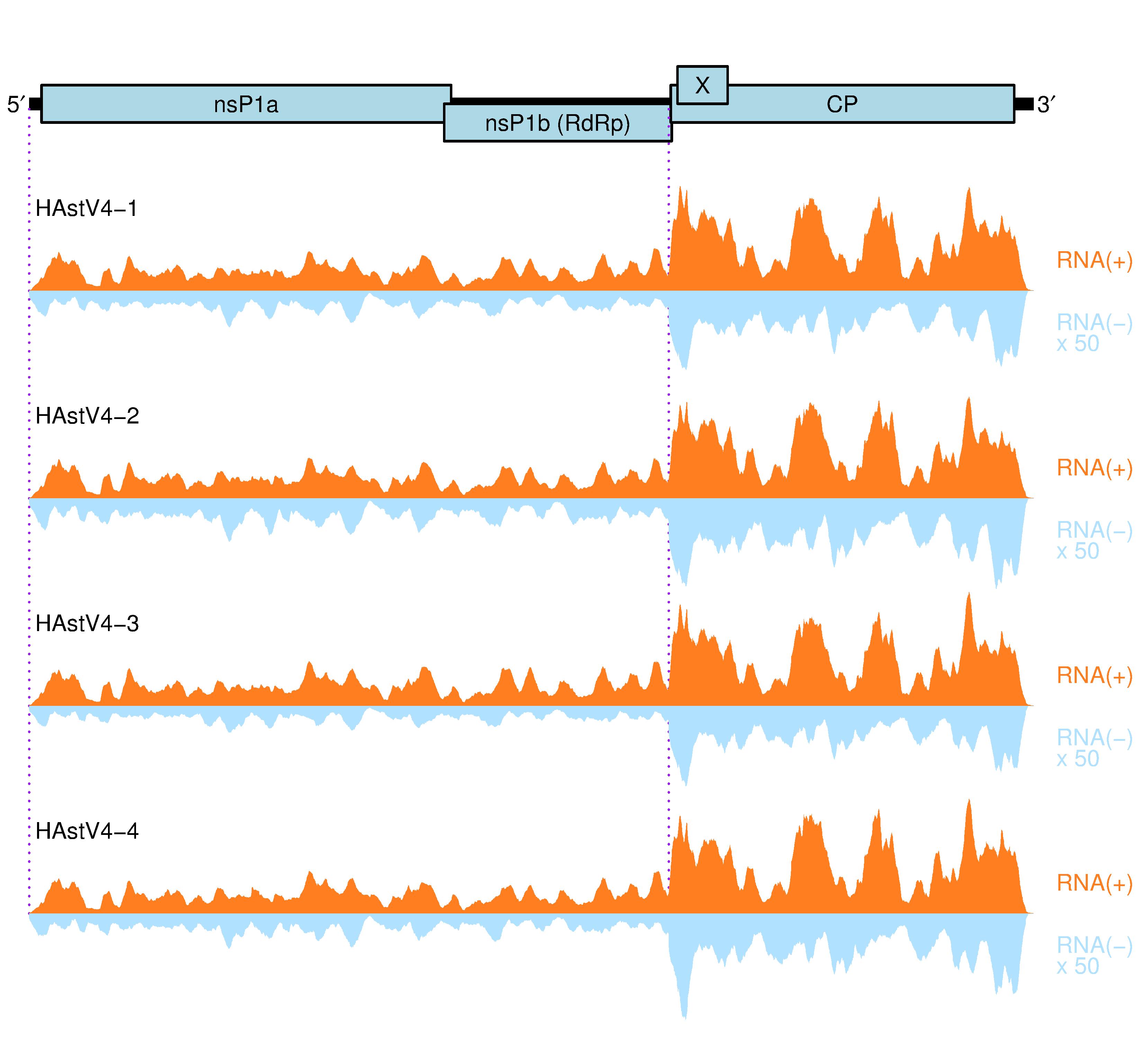

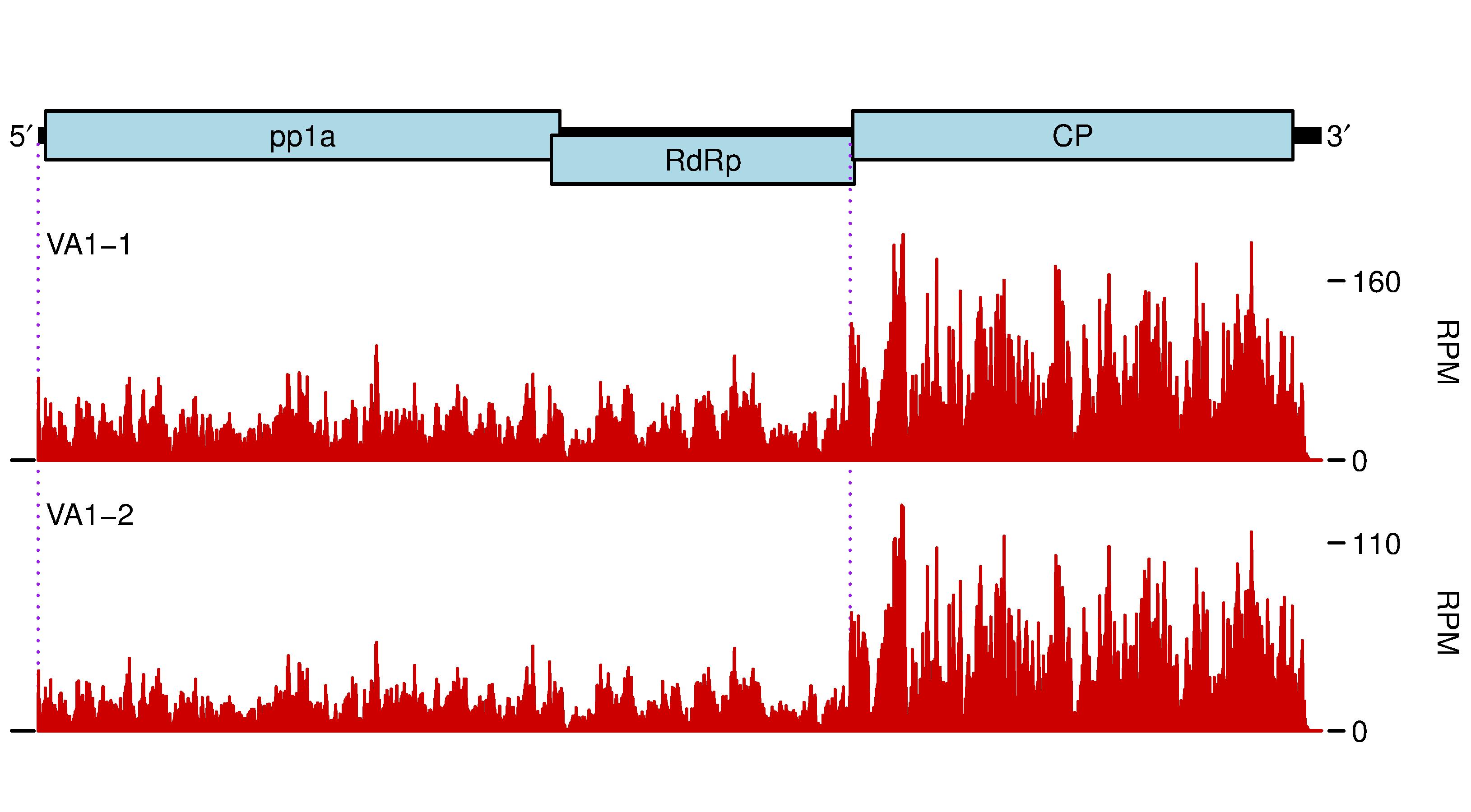

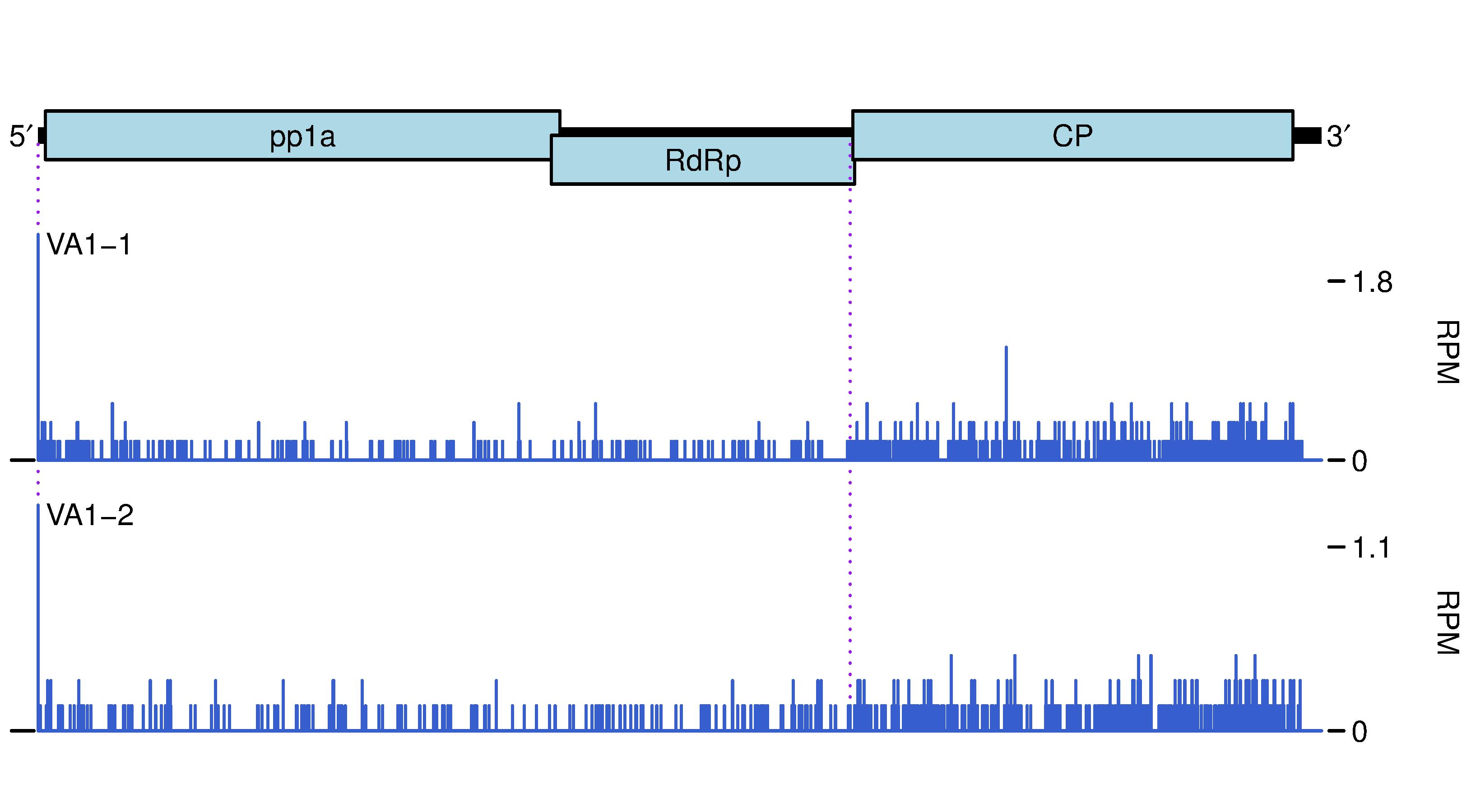
**

**
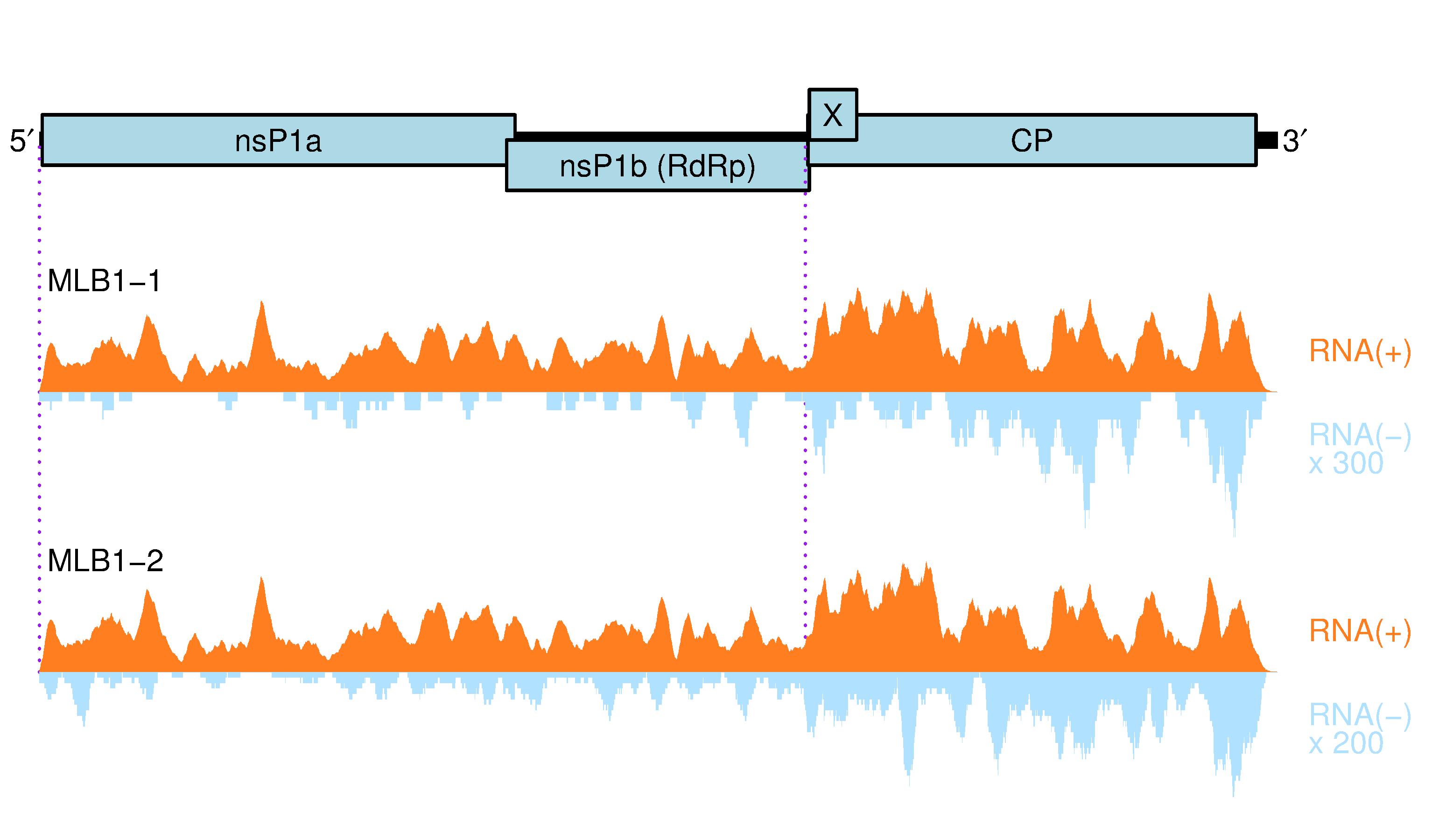
Supplementary Figure S15. Total coverage of vRNA(+) and vRNA(−).** Huh7.5.1 cells were infected with MLB1 astrovirus at MOI 5 and harvested at 24 hpi in duplicate. Fragments were mapped to vRNA(+) or vRNA(−), and total depth of coverage summed. The y-axis scale is arbitrary but vRNA(−) coverage depth is scaled relative to vRNA(+) coverage depth by the indicated factor to aid visualization.

**Supplementary Figure S16. Total coverage of vRNA(+) and vRNA(−).** Huh7.5.1 cells were infected with MLB2 astrovirus at MOI 5 and harvested at 24 hpi in duplicate. Fragments were mapped to vRNA(+) or vRNA(−), and total depth of coverage summed. The y-axis scale is arbitrary but vRNA(−) coverage depth is **
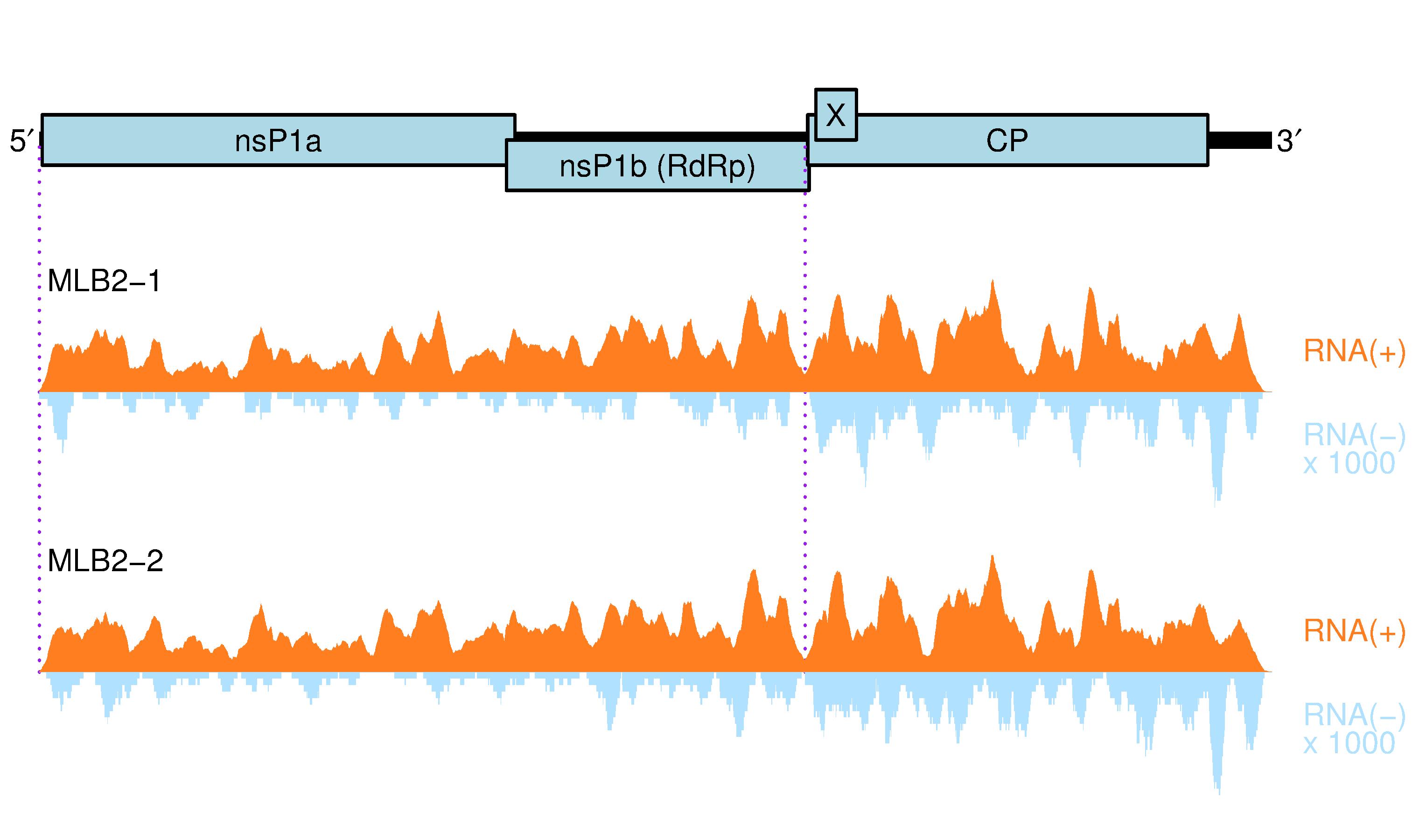
**scaled relative to vRNA(+) coverage depth by the indicated factor to aid visualization.

**
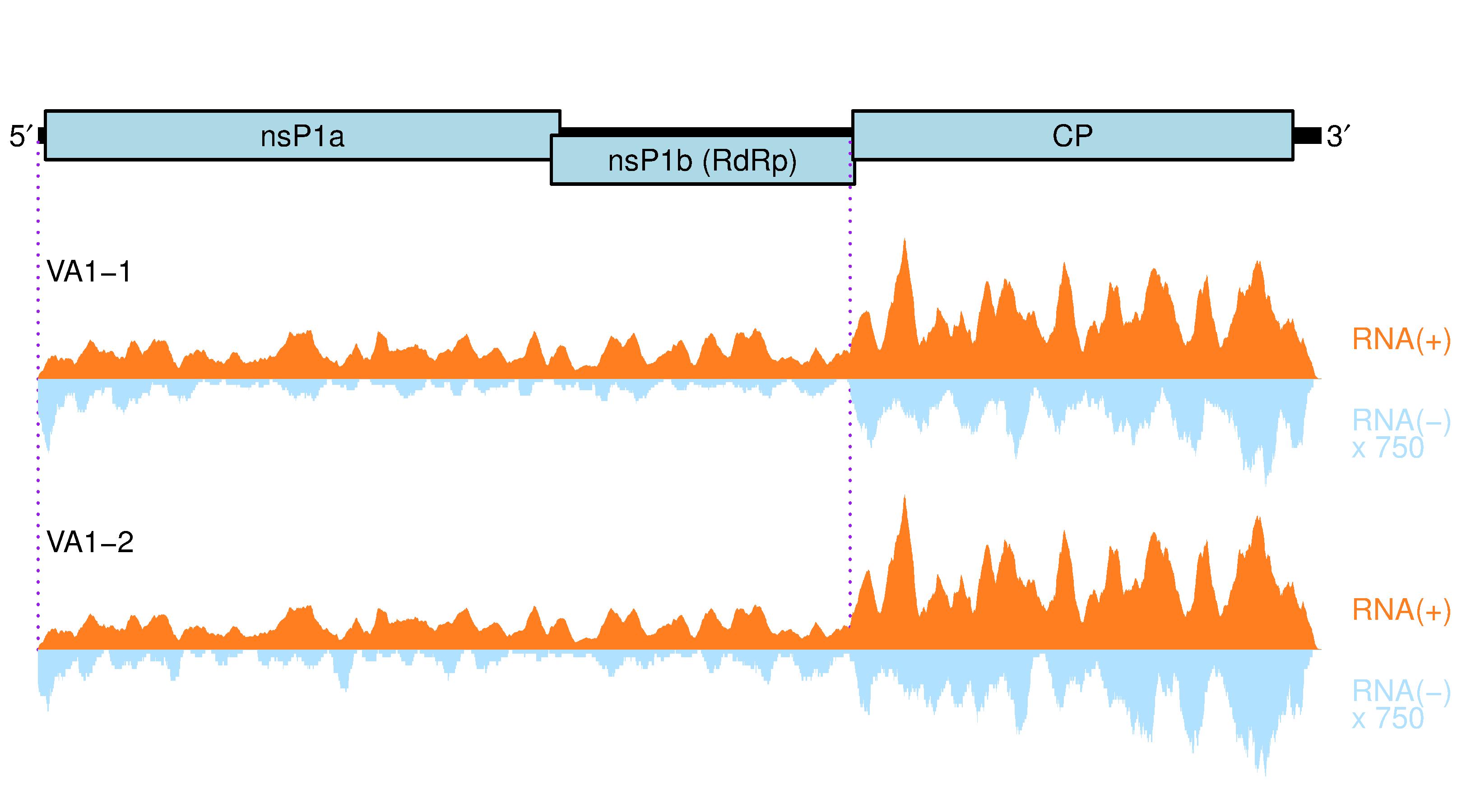
Supplementary Figure S17. Total coverage of vRNA(+) and vRNA(−).** Caco-2 cells were infected with VA1 astrovirus at MOI 5 and harvested at 24 hpi in duplicate. Fragments were mapped to vRNA(+) or vRNA(−), and total depth of coverage summed. The y-axis scale is arbitrary but vRNA(−) coverage depth is scaled relative to vRNA(+) coverage depth by the indicated factor to aid visualization.

**Supplementary Figure S18. Histograms showing positions of 5′ ends of vRNA(+) fragments.** Caco-2 cells were infected with HAstV4 at MOI 5 and harvested at 24 hpi in quadruplicate. Counts are normalized to fragments per million fragments mapped to vRNA(+) or host mRNA(+) (FPM). Histograms show 5′ ends of positive-sense fragments.

**
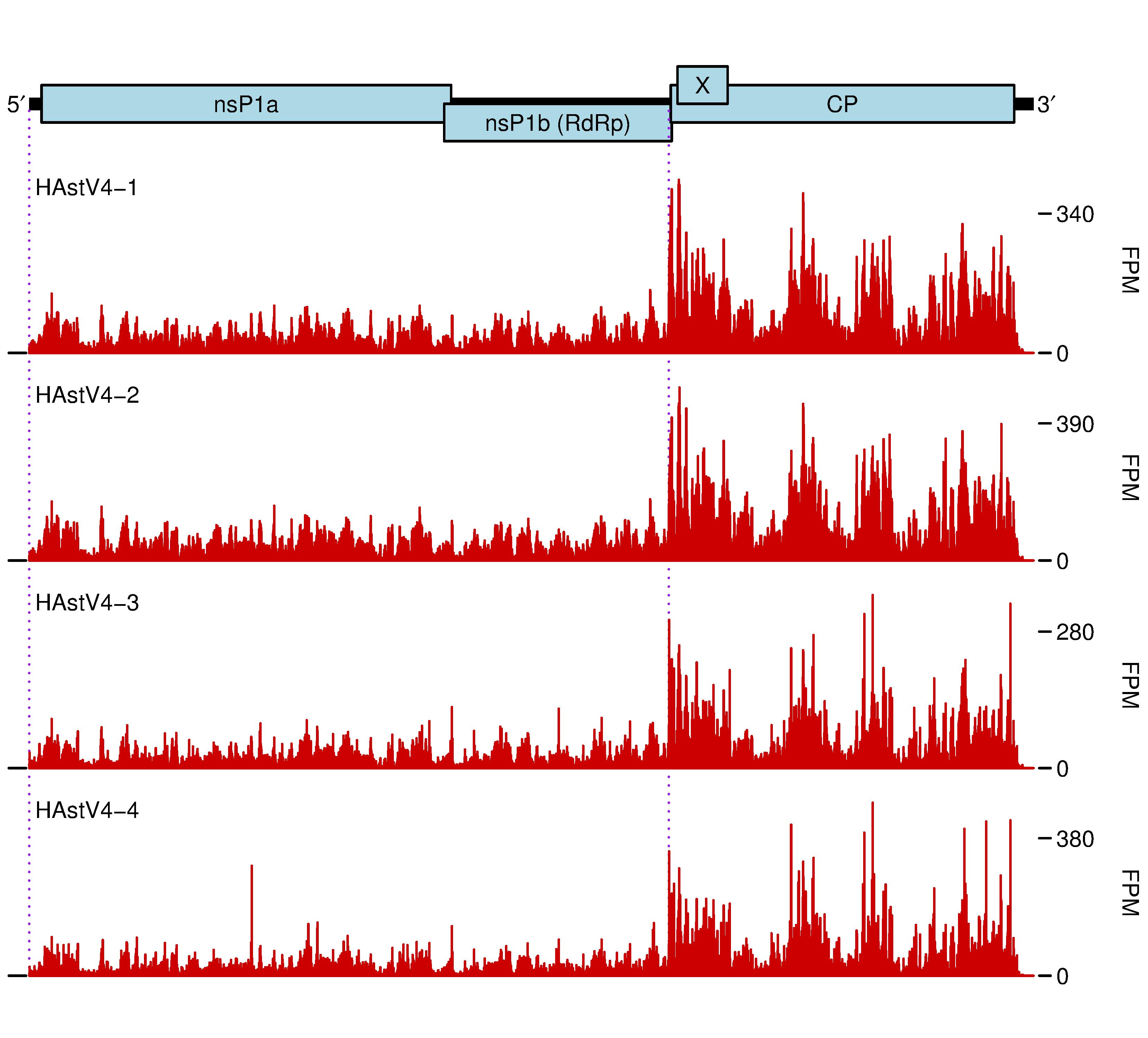
**

**
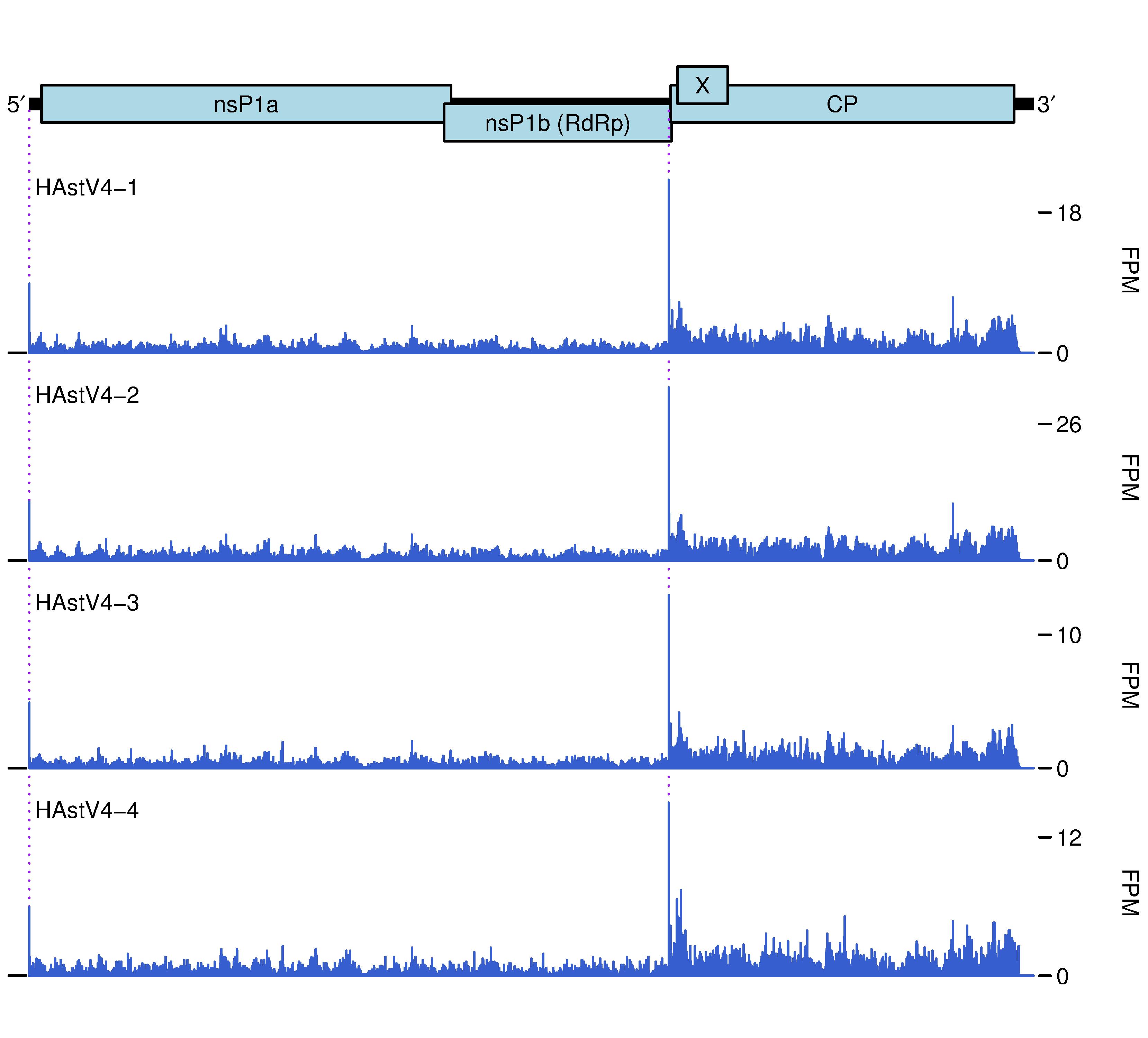
Supplementary Figure S19. Histograms showing positions of 3′ ends of vRNA(−) fragments.** Caco-2 cells were infected with HAstV4 at MOI 5 and harvested at 24 hpi in quadruplicate. Counts are normalized to fragments per million fragments mapped to vRNA(+) or host mRNA(+) (FPM). Histograms show 3′ ends of negative-sense fragments, corresponding to 5′ ends of the positive-sense reverse complements of the fragments.

**
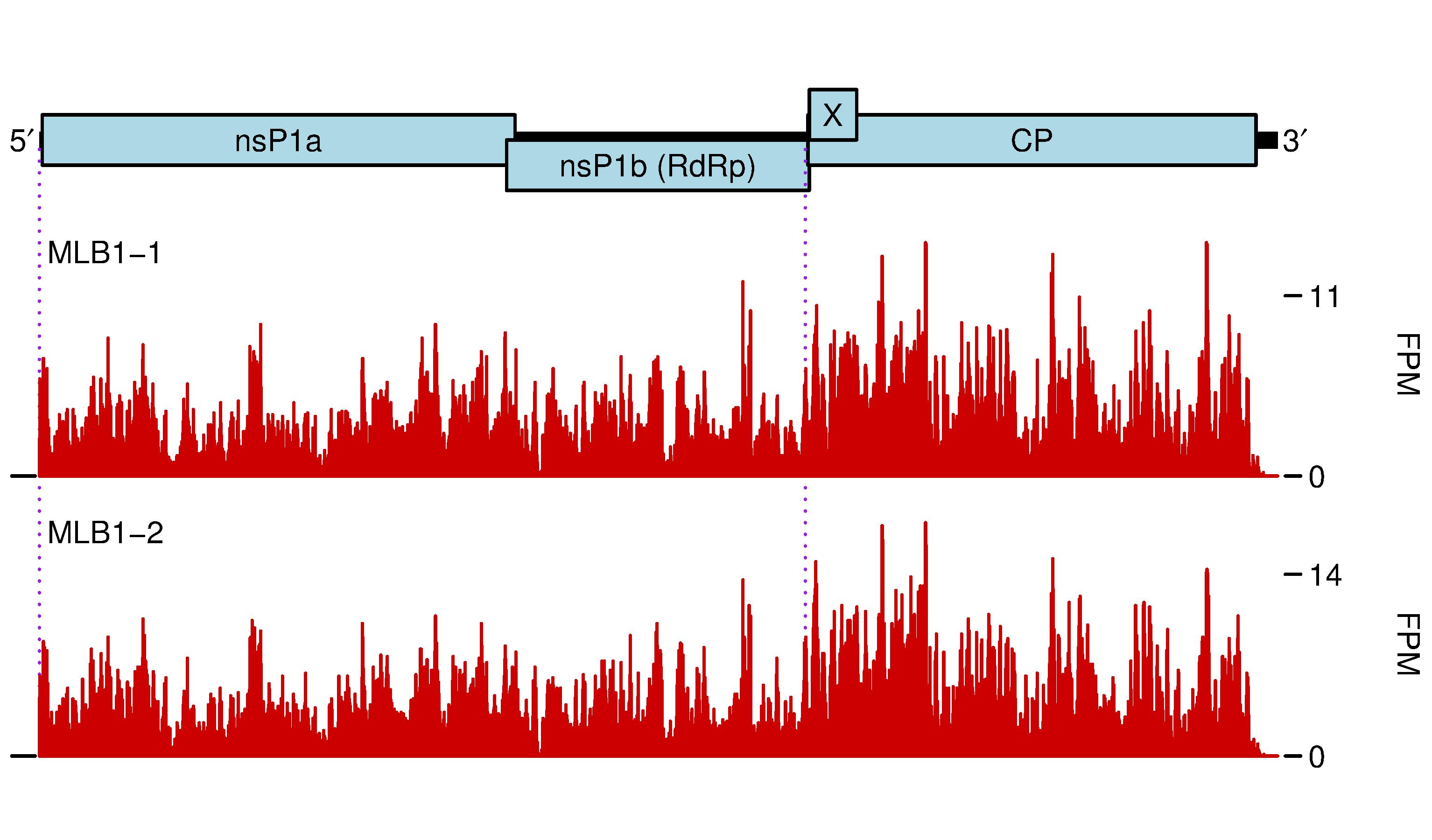
Supplementary Figure S20. Histograms showing positions of 5′ ends of vRNA(+) fragments and 3′ ends of vRNA(−) fragments.** Huh7.5.1 cells were infected with MLB1 astrovirus at MOI 5 and harvested at 24 hpi in duplicate. Counts are normalized to fragments per million fragments mapped to vRNA(+) or host mRNA(+) (FPM). Histograms show 5′ ends of positive-sense fragments (red, upper plots) and 3′ ends of negative-sense fragments, corresponding to 5′ ends of the positive-sense reverse complements of the fragments (blue, lower plots).

**
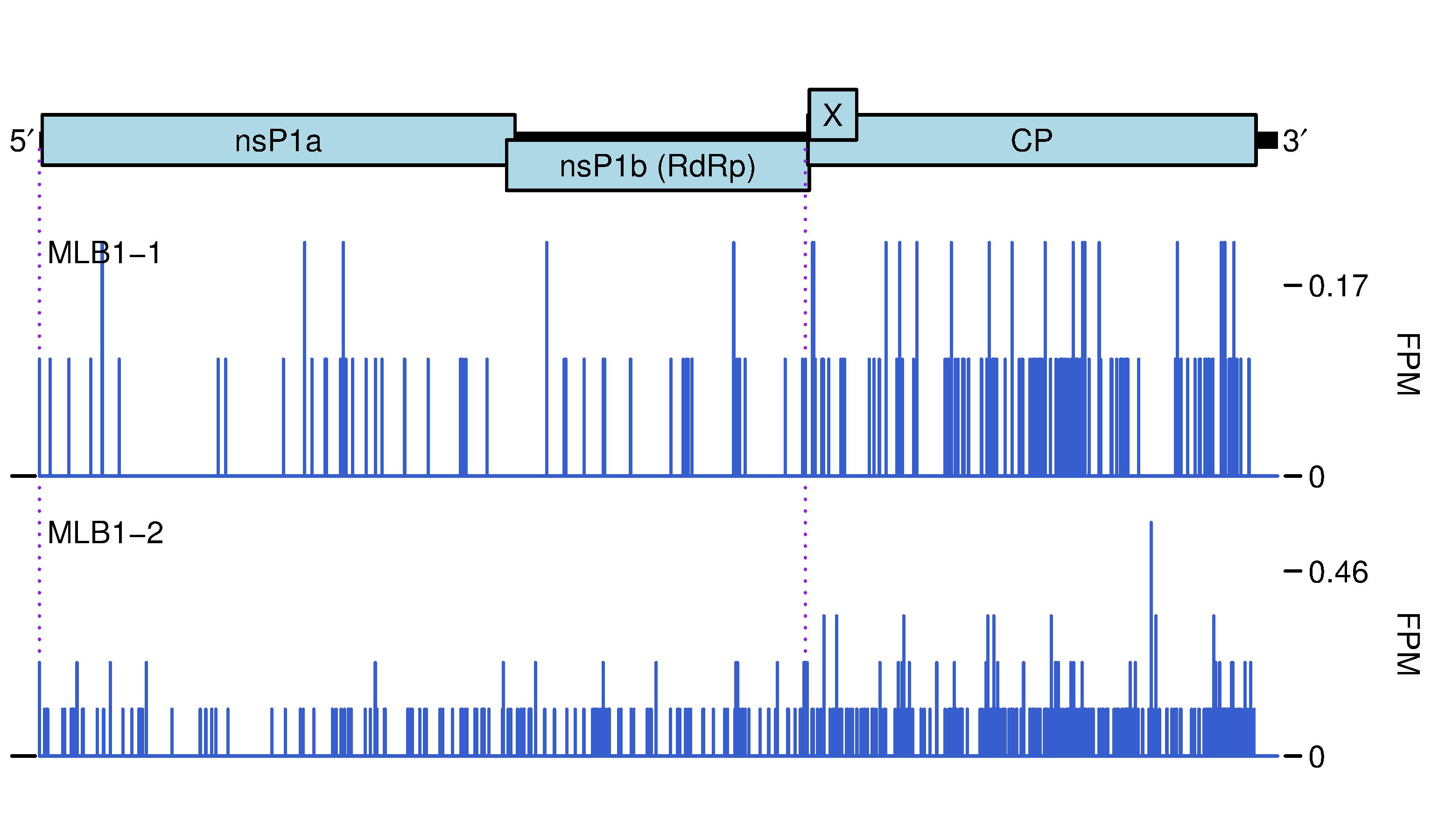
**

**
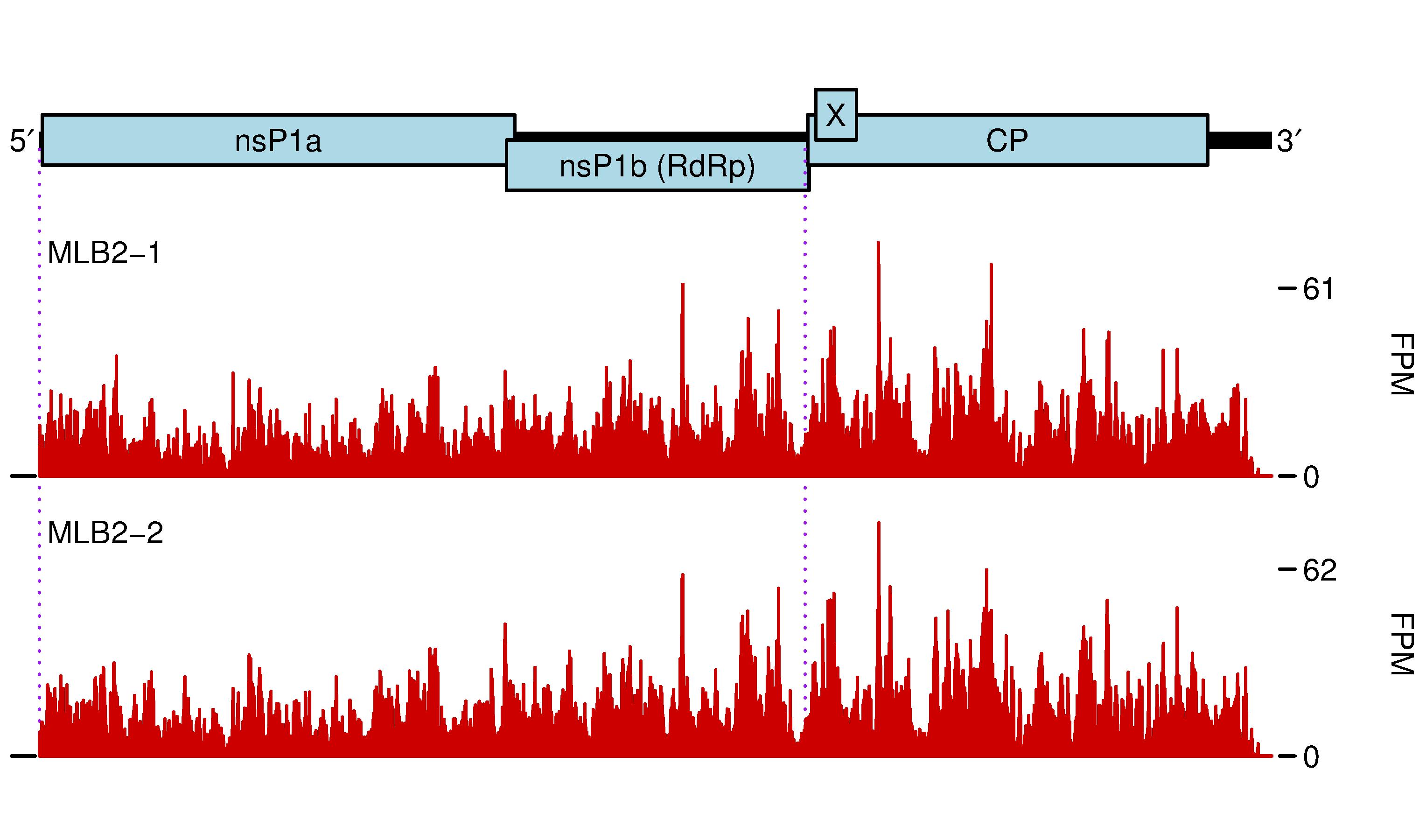
Supplementary Figure S21. Histograms showing positions of 5′ ends of vRNA(+) fragments and 3′ ends of vRNA(−) fragments.** Huh7.5.1 cells were infected with MLB2 astrovirus at MOI 5 and harvested at 24 hpi in duplicate. Counts are normalized to fragments per million fragments mapped to vRNA(+) or host mRNA(+) (FPM). Histograms show 5′ ends of positive-sense fragments (red, upper plots) and 3′ ends of negative-sense fragments, corresponding to 5′ ends of the positive-sense reverse complements of the fragments (blue, lower plots).

**
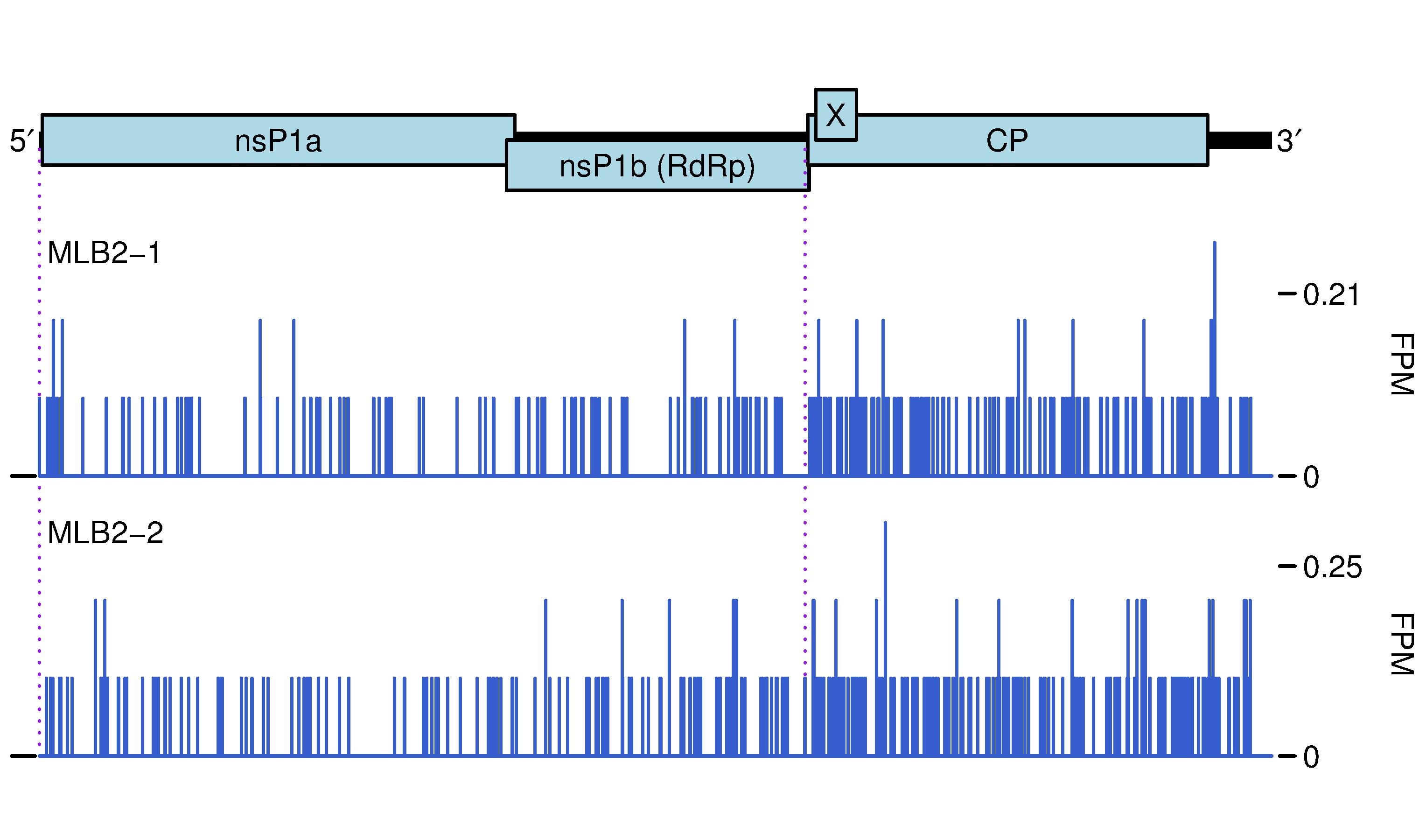
**

**Supplementary Figure S22. Histograms showing positions of 5′ ends of vRNA(+) fragments and 3′ ends of vRNA(−) fragments.** Caco-2 cells were infected with VA1 astrovirus at MOI 5 and harvested at 24 hpi in duplicate. Counts are normalized to fragments per million fragments mapped to vRNA(+) or host mRNA(+) (FPM). Histograms show 5′ ends of positive-sense fragments (red, upper plots) and 3′ ends of negative-sense fragments, corresponding to 5′ ends of the positive-sense reverse complements of the fragments (blue, lower plots).


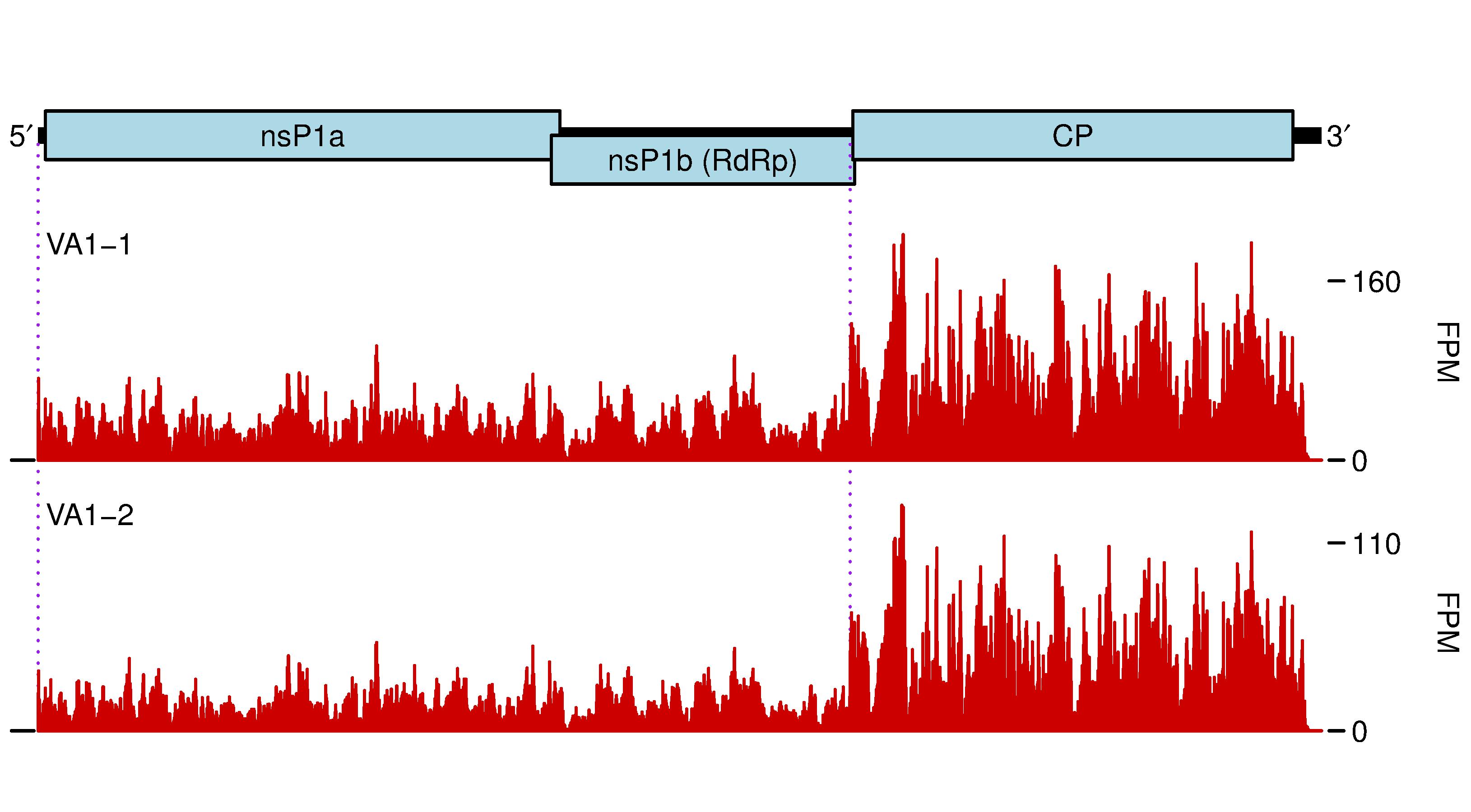


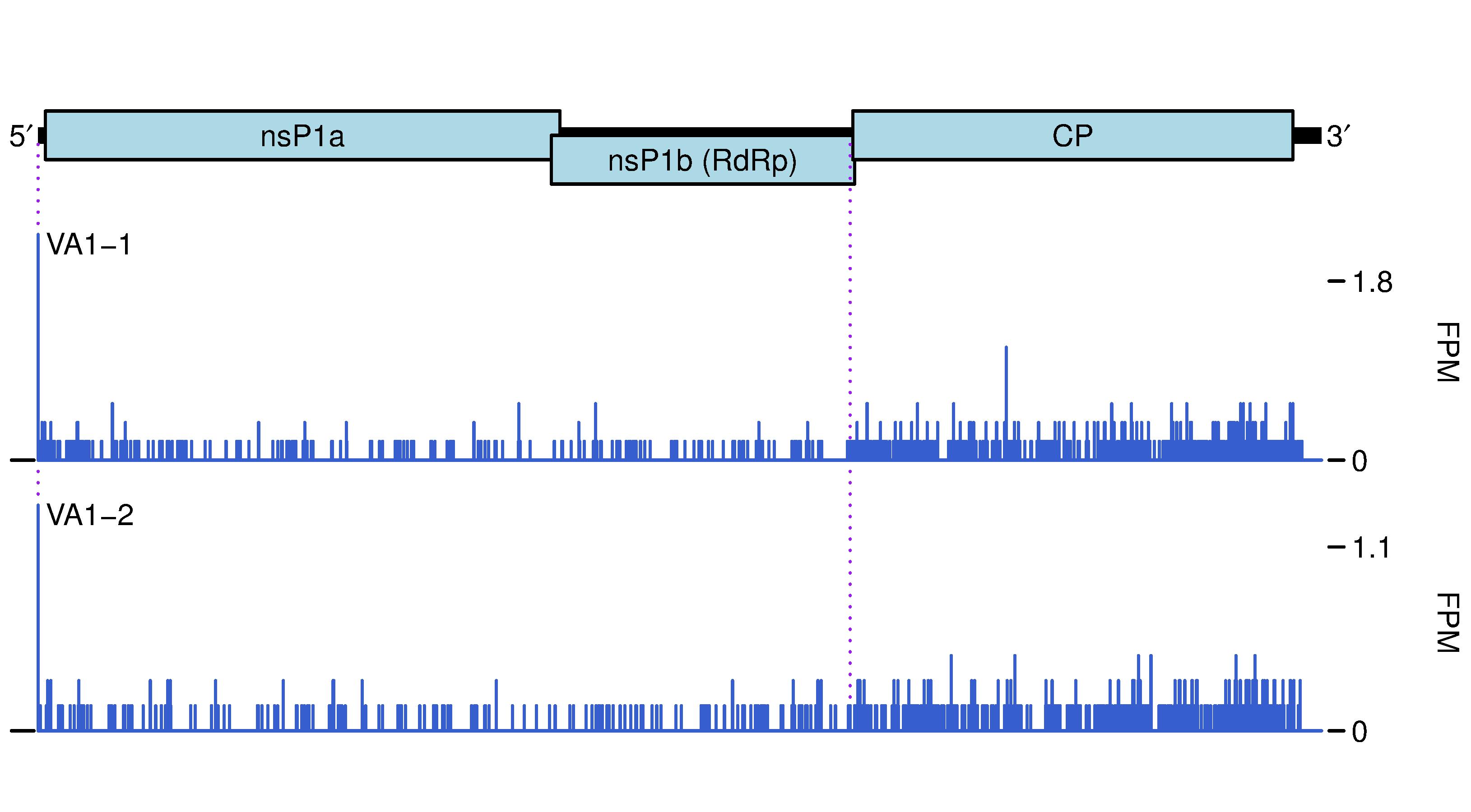


**Supplementary Table S1.** Host and virus read counts for the first high throughput sequencing dataset. Virus = HAstV1. Single-end sequencing. Total read counts are for the raw reads before any quality control or mapping.

| Timepoint | Repeat | Total reads | Host mRNA | vRNA(+) | vRNA(−) |
| --- | --- | --- | --- | --- | --- |
| 6 hpi | 1 | 35391199 | 5831197 | 52486 | 289 |
| 6 hpi | 2 | 41581106 | 6484718 | 67325 | 211 |
| 12 hpi | 1 | 15457347 | 3007362 | 630512 | 421 |
| 12 hpi | 2 | 11749063 | 2098351 | 469602 | 992 |
| 18 hpi | 1 | 16531373 | 2687283 | 1906618 | 11192 |
| 18 hpi | 2 | 24027747 | 3588304 | 2495754 | 17932 |

**Supplementary Table S2.** Host and virus read counts for the second high throughput sequencing dataset. Virus = HAstV1. Paired-end sequencing. Total read counts are the number of raw read pairs before any quality control or mapping. vRNA and mRNA read counts are for deduplicated and mapped read pairs.

| Timepoint | Repeat | Gel slice (nt) | Prot. K | Total read pairs | Host mRNA | vRNA(+) | vRNA(−) |
| --- | --- | --- | --- | --- | --- | --- | --- |
| 18 hpi | 1 | 75–150 | − | 12710966 | 1457826 | 2078280 | 11671 |
| 18 hpi | 2 | 75–150 | − | 9058305 | 1045556 | 1304349 | 1921 |
| 18 hpi | 1 | 75–150 | + | 10050648 | 702968 | 913427 | 22786 |
| 18 hpi | 2 | 75–150 | + | 6608003 | 731704 | 1946024 | 22196 |
| 24 hpi | 1 | 75–150 | − | 8946865 | 1171613 | 1401184 | 1655 |
| 24 hpi | 2 | 75–150 | − | 8280228 | 1053373 | 1215815 | 1726 |
| 24 hpi | 1 | 75–150 | + | 8712935 | 522112 | 722780 | 9717 |
| 24 hpi | 2 | 75–150 | + | 7151543 | 288727 | 470590 | 5297 |
| 18 hpi | 1 | 120–150 | − | 11103758 | 1480799 | 2097240 | 11353 |
| 18 hpi | 2 | 120–150 | − | 11191634 | 1682797 | 1998959 | 2908 |
| 18 hpi | 1 | 120–150 | + | 10759045 | 1008499 | 1259080 | 35844 |
| 18 hpi | 2 | 120–150 | + | 8627940 | 1024741 | 2685722 | 31178 |
| 24 hpi | 1 | 120–150 | − | 5862709 | 963411 | 1161242 | 1264 |
| 24 hpi | 2 | 120–150 | − | 7604984 | 1335713 | 1400545 | 1985 |
| 24 hpi | 1 | 120–150 | + | 6892147 | 509176 | 662140 | 8984 |
| 24 hpi | 2 | 120–150 | + | 11390288 | 662353 | 998886 | 11677 |

**Supplementary Table S3.** Host and virus read counts for the third high throughput sequencing dataset. Paired-end sequencing. Total read counts are the number of raw read pairs before any quality control or mapping. vRNA and mRNA read counts are for mapped read pairs.

| Virus | Timepoint | Repeat | Total reads | Host mRNA | vRNA(+) | vRNA(−) |
| --- | --- | --- | --- | --- | --- | --- |
| HAstV4 | 24 hpi | 1 | 54319472 | 10058210 | 4597902 | 68077 |
| HAstV4 | 24 hpi | 2 | 29725722 | 4651497 | 2858424 | 50542 |
| HAstV4 | 24 hpi | 3 | 52589995 | 8809744 | 1994507 | 25517 |
| HAstV4 | 24 hpi | 4 | 36124894 | 5940483 | 1459639 | 23026 |
| MLB1 | 24 hpi | 1 | 47814255 | 9478754 | 140274 | 199 |
| MLB1 | 24 hpi | 2 | 46267125 | 8458245 | 158913 | 454 |
| MLB2 | 24 hpi | 1 | 66504586 | 10482777 | 690842 | 277 |
| MLB2 | 24 hpi | 2 | 71856494 | 9091993 | 673832 | 331 |
| VA1 | 24 hpi | 1 | 31106709 | 4184063 | 1109417 | 772 |
| VA1 | 24 hpi | 2 | 28990966 | 5872869 | 797653 | 659 |

**Supplementary Table S4.** Raw data for Figure 4B-E. The assay robustness is defined by the WT/GNN ratio (last row).

| Figure | | 4B | | 4C | | 4D | | 4E | |
| --- | --- | --- | --- | --- | --- | --- | --- | --- | --- |
| Replicon | | HAstV1 | HAstV1 | HAstV1 | HAstV1 | MLB2 | MLB2 | MLB2 | MLB2 |
| Cell line | | Huh7.5.1 | BSR | Huh7.5.1 | BSR | Huh7.5.1 | HEK293T | Huh7.5.1 | HEK293T |
| RNA | | G | G | SG | SG | G | G | SG | SG |
| WT | #1 | 1.13E+02 | 1.11E+02 | 9.76E+01 | 9.77E+01 | 8.79E+01 | 9.51E+01 | 7.86E+01 | 1.05E+02 |
|  | #2 | 8.93E+01 | 9.70E+01 | 1.03E+02 | 1.03E+02 | 1.08E+02 | 1.05E+02 | 1.22E+02 | 9.04E+01 |
|  | #3 | 9.75E+01 | 9.21E+01 | 9.91E+01 | 9.94E+01 | 1.04E+02 | 1.00E+02 | 9.93E+01 | 1.05E+02 |
| GNN | #1 | 3.50E+01 | 1.12E+01 | 1.42E-01 | 1.24E-01 | 2.74E+01 | 4.09E+01 | 1.01E+00 | 2.27E-01 |
|  | #2 | 3.91E+01 | 7.71E+00 | 1.83E-01 | 1.17E-01 | 1.95E+01 | 3.45E+01 | 6.89E-01 | 2.12E-01 |
|  | #3 | 3.41E+01 | 1.28E+01 | 1.53E-01 | 1.12E-01 | 3.52E+01 | 3.13E+01 | 1.06E+00 | 2.05E-01 |
| gCGAA | #1 | 5.51E+01 | 1.70E+01 | 7.17E+01 | 8.37E+01 | 2.60E+01 | 2.52E+01 | 4.78E+01 | 7.75E+00 |
|  | #2 | 6.16E+01 | 1.82E+01 | 7.97E+01 | 9.11E+01 | 2.19E+01 | 2.41E+01 | 4.11E+01 | 9.38E+00 |
|  | #3 | 5.54E+01 | 1.91E+01 | 8.40E+01 | 9.24E+01 | 2.00E+01 | 3.38E+01 | 4.08E+01 | 1.10E+01 |
| gCCUA | #1 | 8.76E+01 | 5.77E+01 | 8.96E+01 | 1.13E+02 | 2.23E+01 | 5.57E+01 | 6.15E+01 | 2.16E+01 |
|  | #2 | 9.54E+01 | 4.64E+01 | 1.03E+02 | 9.51E+01 | 1.72E+01 | 5.20E+01 | 4.46E+01 | 2.40E+01 |
|  | #3 | 7.09E+01 | 5.45E+01 | 8.54E+01 | 1.06E+02 | 2.14E+01 | 5.63E+01 | 5.58E+01 | 3.13E+01 |
| gCCAU | #1 | 8.44E+01 | 8.43E+01 | 1.18E+02 | 1.46E+02 | 2.29E+01 | 4.22E+01 | 2.25E+01 | 4.16E+01 |
|  | #2 | 7.03E+01 | 7.60E+01 | 1.30E+02 | 1.39E+02 | 2.10E+01 | 3.93E+01 | 1.93E+01 | 3.28E+01 |
|  | #3 | 7.47E+01 | 7.10E+01 | 1.07E+02 | 1.44E+02 | 3.12E+01 | 3.51E+01 | 2.88E+01 | 1.68E+01 |
| sgCGAA | #1 | 2.27E+02 | 1.75E+02 | 9.95E-01 | 1.42E+00 | 7.75E+01 | 6.23E+01 | 2.32E+00 | 3.36E-01 |
|  | #2 | 2.57E+02 | 1.95E+02 | 1.78E+00 | 1.54E+00 | 5.96E+01 | 5.00E+01 | 1.67E+00 | 3.33E-01 |
|  | #3 | 2.34E+02 | 2.17E+02 | 1.16E+00 | 1.56E+00 | 7.06E+01 | 5.83E+01 | 2.10E+00 | 3.58E-01 |
| sgCCUA | #1 | 1.61E+02 | 1.54E+02 | 1.33E+01 | 5.76E+00 | 4.74E+01 | 6.62E+01 | 8.19E+00 | 2.69E+00 |
|  | #2 | 2.09E+02 | 1.45E+02 | 1.30E+01 | 4.76E+00 | 2.75E+01 | 8.41E+01 | 5.06E+00 | 1.88E+00 |
|  | #3 | 1.87E+02 | 1.59E+02 | 1.33E+01 | 5.81E+00 | 3.28E+01 | 6.34E+01 | 7.00E+00 | 1.44E+00 |
| sgCCAU | #1 | 1.47E+02 | 6.74E+01 | 2.46E+01 | 5.94E+00 | 2.80E+01 | 7.04E+01 | 2.73E+00 | 1.15E+00 |
|  | #2 | 1.27E+02 | 5.64E+01 | 2.20E+01 | 5.74E+00 | 3.24E+01 | 6.91E+01 | 2.60E+00 | 1.23E+00 |
|  | #3 | 1.30E+02 | 5.47E+01 | 2.15E+01 | 5.87E+00 | 3.55E+01 | 7.52E+01 | 2.89E+00 | 1.25E+00 |
| WT/GNN | | 2.77 | 9.46 | 627.22 | 851.37 | 3.65 | 2.81 | 108.76 | 465.68 |
